# Cyanobacteria as Natural Therapeutics and Pharmaceutical Potential: Role in Antitumor Activity and as Nanovectors

**DOI:** 10.3390/molecules26010247

**Published:** 2021-01-05

**Authors:** Hina Qamar, Kashif Hussain, Aishwarya Soni, Anish Khan, Touseef Hussain, Benoît Chénais

**Affiliations:** 1 Interdisciplinary Biotechnology Unit, Aligarh Muslim University, Aligarh 202002, India; hina.dna@rediffmail.com; 2 Pharmacy Section, Gyani Inder Singh Institute of Professional Studies, Dehradun 248003, India; kashif@gisips.com; 3 School of Pharmacy, Glocal University, Saharanpur 247121, India; 4 Department of Biotechnology, Deenbandhu Chhotu Ram University of Science and Technology, Murthal, Sonepat 124001, India; aishwaryasoni32@gmail.com; 5 Centre for Biotechnology, Maharshi Dayanand University, Rohtak 124001, India; anishkhan.rs.biotech@mdurohtak.ac.in; 6 Department of Botany, Aligarh Muslim University, Aligarh 202002, India; 7 EA 2160 Mer Molécules Santé, Le Mans Université, F-72085 Le Mans, France

**Keywords:** cyanobacteria, bioactivity, natural compounds, anti-tumor, cytotoxicity, secondary metabolites, nanoparticles

## Abstract

Cyanobacteria (blue-green microalgae) are ubiquitous, Gram-negative photoautotrophic prokaryotes. They are considered as one of the most efficient sources of bioactive secondary metabolites. More than 50% of cyanobacteria are cultivated on commercial platforms to extract bioactive compounds, which have bene shown to possess anticancer activity. The chemically diverse natural compounds or their analogues induce cytotoxicity and potentially kill a variety of cancer cells via the induction of apoptosis, or altering the activation of cell signaling, involving especially the protein kinase-C family members, cell cycle arrest, mitochondrial dysfunctions and oxidative damage. These therapeutic properties enable their use in the pharma and healthcare sectors for the betterment of future generations. This review provides a baseline overview of the anti-cancerous cyanobacterial bioactive compounds, along with recently introduced nanomaterials that could be used for the development of new anticancer drugs to build a healthy future for mankind.

## 1. Introduction

**Figure 1:**
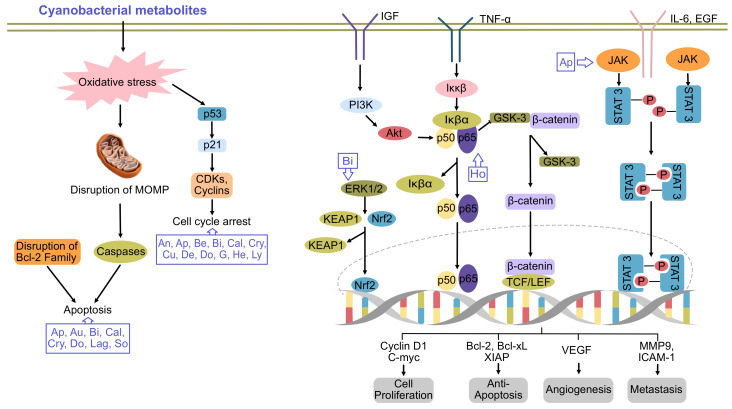
Major pathways of cancer inhibited by cyanobacterial metabolites. An, ankarholide; Ap, apratoxin; Au, aurilide; Be, belamide A; Bi, bisebromoamide; Cal, calothrixin A; Cry, cryptophycin; Cu, curacin A; De, desmethoxymajusculamide; Do, dolastatins; G, grassystatin; He, heterochlorin; Ho, hoiamide; Lag, lagunamide; Lyn, lyngbyabellin; So, somocystinamide A. Adpated and enhanced from [7].

Cyanobacteria (blue-green microalgae) are ubiquitous Gram-negative photoautotrophic prokaryotes, for which evidence they existed 3.3–3.5 billion years ago was found [1]. They exist in the environment as unicellular, filamentous or colonial forms surrounded by a mucilaginous sheath [2]. Owing to their ubiquitous nature and the vast diversity of their metabolites, they have several applications in the biomedical field. During the last few decades, the emergence of new cancers and their developing resistance to available drugs has led to chemotherapeutic failure. Thus, the identification of new biologically active compounds is immediately required for the development of new drugs. In this regard, the medicinal properties of cyanobacteria have been explored. To date, several secondary metabolites have been isolated from the members of oscillatoriales (49%), followed by nostocales (26%), chroococcales (16%), pleurocapsales (6%) and stigonematales (4%) [2,3]. The capacity of cyanobacteria to integrate non-ribosomal peptide synthetases with polyketide synthases enables them to produce biologically active and chemically diverse compounds, such as cyclic alkaloids, peptides, depsipeptides, cyclic depsipeptides, fatty acid, lipopeptides, swinholides and saccharides [4]. Back in 1500 BC, *Nostoc* sp. was first used to treat gout, fistula and different types of cancers [5]. However, more focused research utilizing modern technologies was started in this field in the 1990s. Several cyanobacteria species are cultivated in commercial platforms to extract bioactive compounds, which have been shown to possess anticancer activity and potentially kill a variety of cancer cells via the induction of apoptosis or altering the activation of cell signaling [4,6] (Figure 1).

## 2. Anticancer Potential of Bioactive Compounds from Cyanobacteria

### 2.1. Ankaraholide A

**Figure 2:**
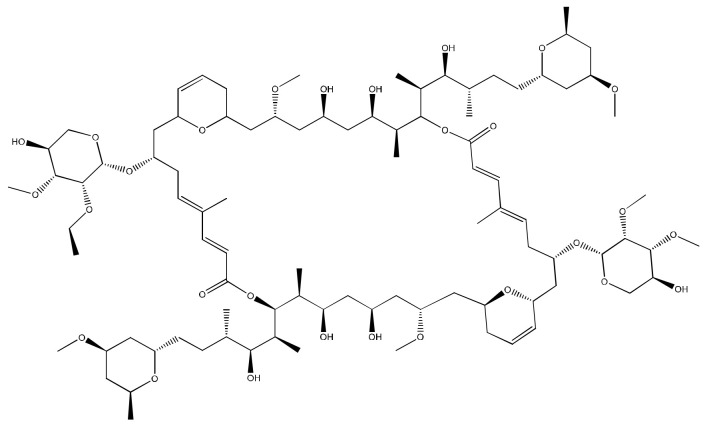
Chemical structure of Ankaraholide A.

Ankaraholide A (Figure 2), a glycosylated swinholide isolated from *Geitlerinema*, repressed proliferation in the mouse neuroblastoma cell line Neuro-2a, the human lung cancer cell line NCI-H460, and the human melanoma cell line MDA-MB-435, with IC_50_ values of 119 nM, 262 nM and 8.9 nM, respectively (Table 1). Furthermore, in the rat non-tumoral myoblast A-10 cells, it caused a complete loss of the filamentous (F)-actin at the 30 nM and 60 nM concentrations [8].

### 2.2. Apratoxin

**Figure 3:**
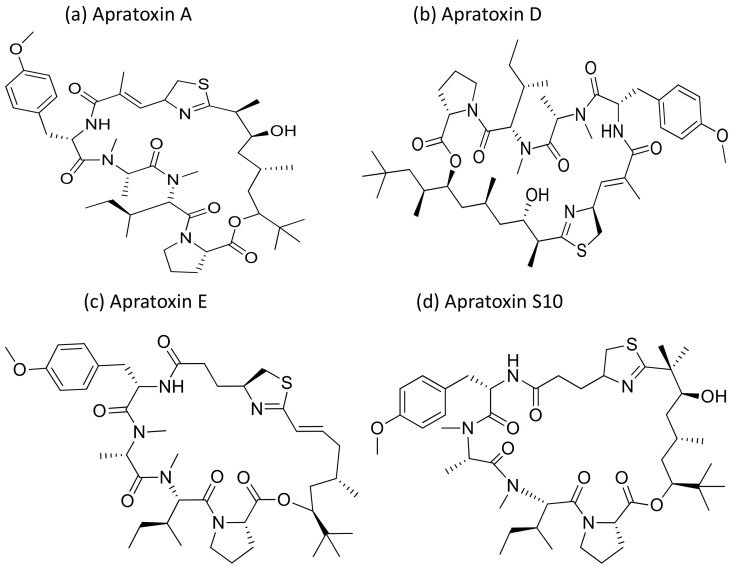
Chemical structures of apratoxins.

Apratoxin, a cyclic depsipeptide isolated from *Micronesia* and *Lyngbya* sp., is a potent cytotoxic marine natural product that showed anti-cancerous activity against various cancer cell lines (Table 1). Apratoxin has antiproliferative effects via the induction of G1 cell cycle arrest and apoptotic cascade [9]. Furthermore, it inhibits the Janus kinase (JAK)/signal transducer and activator of transcription (STAT) signaling pathway by downregulating the interleukin (IL)-6 molecule signal transducer (gp130) and reversibly inhibiting the secretory pathway by preventing cotranslational translocation early in the secretory pathway [10]. Apratoxin A (Figure 3), isolated from *Lyngbya* sp., induces cytotoxicity on adenocarcinoma cells, and showed cytotoxicity in the nanomolar range when tested against HT29 (colorectal adenocarcinoma), HeLa (cervix adenocarcinoma) and U2OS (osteosarcoma) cancer cell lines. The IC_50_ value ranges from 0.36 to 0.52 nM for in vitro studies while, for in vivo studies it was only marginally active against a colon tumor, and was ineffective against a mammary tumor [11]. Furthermore, apratoxin also affects the apoptosis of cancer cells by downregulating receptors and the associated growth ligand. A hybrid synthetic molecule that combines both apratoxins A and E has been synthesized with improved in vivo antitumor properties. It downregulated receptor tyrosine kinases and vascular endothelial growth factor A (VEGF-A) in a colorectal tumor xenograft model [12]. Oxazoline, a synthetic analogue of apratoxin A, inhibits the function of heat shock protein 90 (Hsp90) and promotes chaperone-mediated autophagy [13]. The in vivo pathological studies against the pancreatic tumor revealed that the Sec 61 complex is the molecular target of apratoxin A [14]. Recently, apratoxin S10 (Apra S10, an apratoxin A analogue) has been developed, which induced promising antitumor effects in the pancreatic cancer model by exhibiting the downregulation of multiple receptor tyrosine kinases and inhibiting several growth factors and cytokine secretion [15]. Moreover, apratoxin D revealed strong cytotoxicity, with an IC_50_ value of 2.6 nM against NCI-H460 lung cancer cells [16].

### 2.3. Aurilide

**Figure 4:**
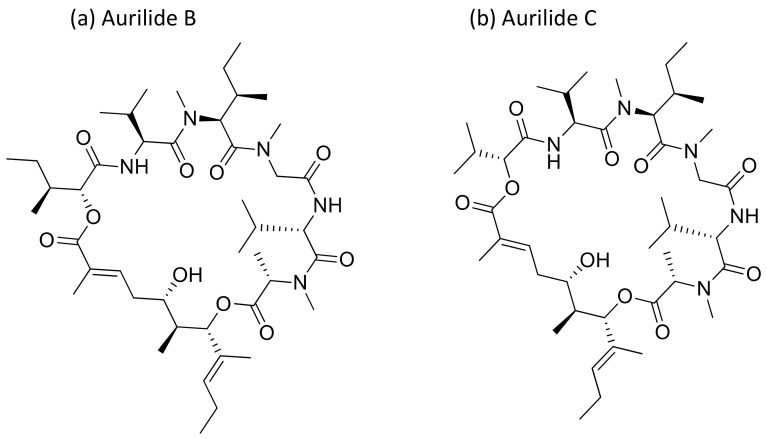
Chemical structure of aurilide analogs.

Aurilide, a cyclic depsipeptide isolated from *Dolabella auricularia,* showed cytotoxicity ranging from picomolar (pM) to nanomolar (nM) concentrations against several cancer cell lines (Table 1) [17]. It encourages mitochondrial-induced apoptosis by selectively binding to prohibitin 1 (PHB1) in the mitochondria and activating the proteolytic processing of optic atrophy 1 (OPA1) [18]. Aurilides B and C (aurilide analogues, Figure 4) showed in vitro cytotoxicity against an NCI-H460 human lung tumor and the neuro-2a mouse neuroblastoma cell lines, with LC_50_ values ranging from 0.01 to 0.13 µM [17]. Aurilide B exhibits potent cytotoxicity against human renal, leukemia and prostate cancer cell lines from the NCI-60 panel, a panel of 60 different human tumor cell lines of different tissue origins, with an average growth inhibition (GI_50_) value of less than 10 nM [17].

### 2.4. Bisebromoamide

**Figure 5:**
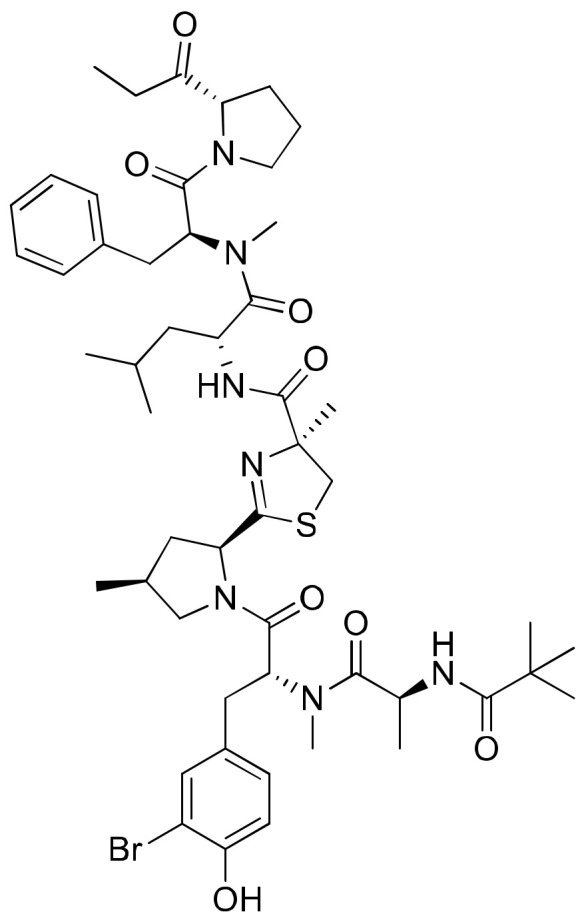
Chemical structure of bisebromoamide.

Bisebromoamide (Figure 5), a linear peptide isolated from an Okinawan strain of *Lyngbya* sp., contains a unique N-methyl-3-bromotyrosine, a modified 4-methylproline, a 2-(1-oxopropyl) pyrrolidine and an N-pivalamide unit. Based on cell morphological profiling analysis, bisebromoamide was identified as an actin filament stabilizer [19]. It revealed cytotoxicity against HeLa S3 cells with an IC_50_ value of 0.04 µg/mL, and exhibited 50% growth inhibition against a panel of 39 human cancer cell lines with a GI_50_ value of 40 nM (Table 1). It exhibited strong protein kinase inhibition by phosphorylating extracellular signal-regulated protein kinase (ERK) in normal rat kidney (NRK) cells via platelet-derived growth factor (PDGF) stimulation at a 0.1–10 µM concentration [20]. However, studies have shown that bisebromoamide and its synthetic analogs, whose stereochemistry reveals the presence of a methylthiazoline moiety and a methyl group at the 4-methylproline unit, do not significantly influence cytotoxicity against HeLa S3 cancer cells [21]. Bisebromoamide inhibited the phosphorylation of ERK and AKT (protein kinase) enzymes when tested against human renal carcinoma cell lines 769-P and 786-O. The IC_50_ values for 769-P and 786-O cells were 1.63 µM and 2.11 µM, respectively [22]. In another study, it was shown to induce apoptosis through ERK and mTOR inhibitions in renal cancer cells [23].

### 2.5. Biselyngbyaside

**Figure 6:**
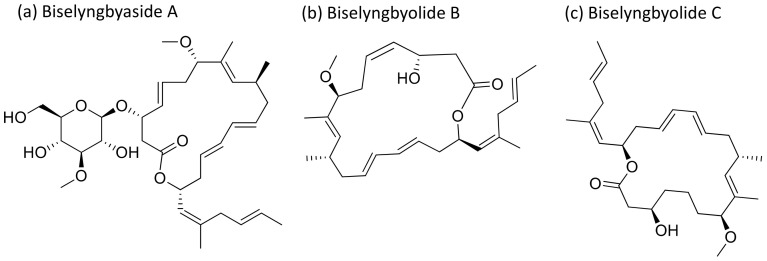
Chemical structure of biselyngbiasides.

Biselyngbyaside A (Figure 6), a glycomacrolide isolated from *Lyngbya* sp., exhibits cytotoxicity against HeLa S3 cells with an IC_50_ value of 0.1 μg/mL [24]. Moreover, Biselyngbyolide B, C, E and F have an antiproliferative effect in HeLa and HL-60 cells, and biselyngbyolide C was found to trigger endoplasmic reticulum (ER) stress and apoptosis in HeLa cells [25] (Table 1).

### 2.6. Borophycin

**Figure 7:**
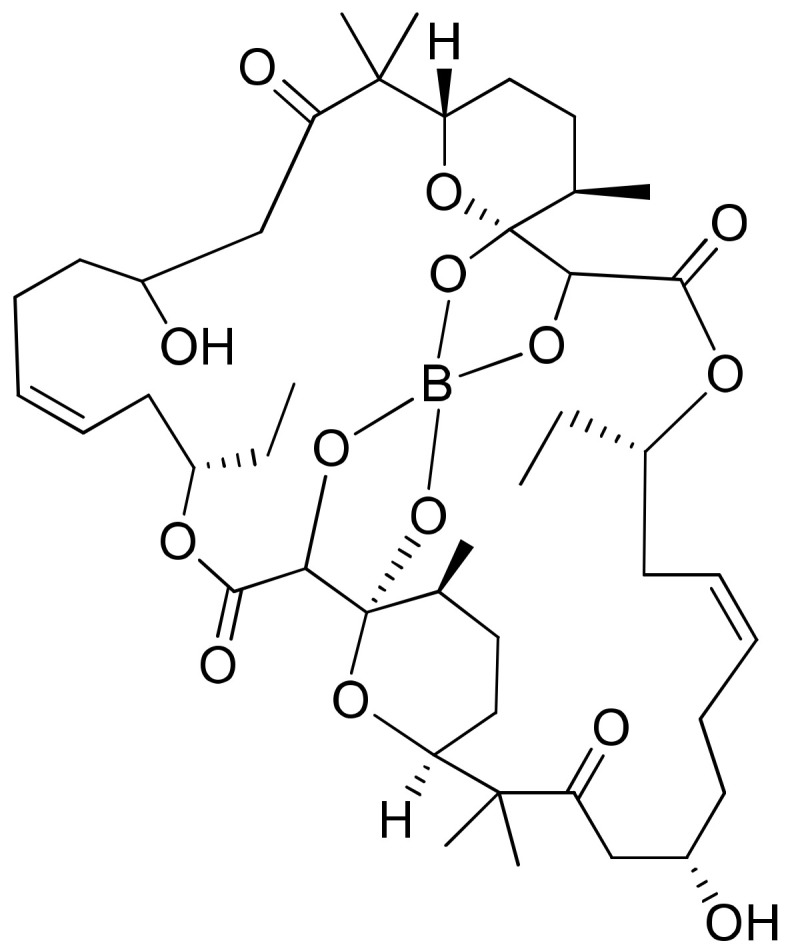
Chemical structure of borophycin.

Borophycin (boron-containing metabolite) is an acetate-derived polyketide (Figure 7) isolated from *Nostoc linckia* and *N. spongiaeforme* var. tenue, which exhibits potent cytotoxicity against human colorectal adenocarcinoma (LoVo) and human cervical adenocarcinoma (KB) cell lines [26] (Table 1).

### 2.7. Calothrixin

**Figure 8:**
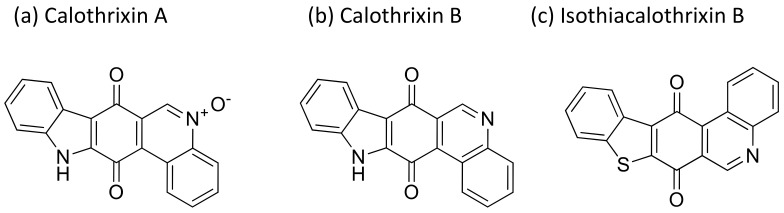
Chemical structure of calothrixins.

Calothrixins are quinone-based bioactive molecules (Figure 8) isolated from *Calothrix* that possess potent antiproliferative activity against several cancer cell lines [27]. Calothrixins A, an indolophenanthridine isolated from *Calothrix*, induced apoptosis and cell cycle arrest in the G2/M phase of several human cancer cell lines (Table 1). It has shown antitumor activity against human HeLa cancer cells at nanomolar concentrations [28]. Besides this, calothrixin B displayed antiproliferative activity against the HCT-116 colon cancer cell line with an IC_50_ value of 0.32 µM [29]. Recently, a series of calothrixin B analogs have been synthesized that inhibit cancer cell growth by extensive DNA damage followed by apoptotic cell death. They have shown cytotoxicity against the NCI-H460 cell line with a GI_50_ of 1 nM [30]. Another analog of calothrixin B, isothiacalothrixin B, exhibits cytotoxicity against human colon cancer cells in vitro by inducing irreversible DNA damage, and causes apoptosis. It was found to be a promising anti-cancer agent that caused the strong arrest of the cells in the S and G2/ M phases of the cell cycle in the HCT116 cell line [31].

### 2.8. Carmaphycins

**Figure 9:**
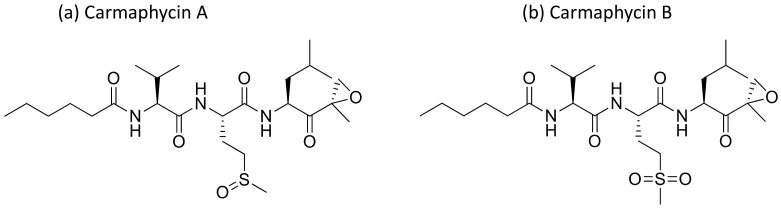
Chemical structure of carmaphycins.

Carmaphycins A and B (Figure 9), isolated from *Symploca* sp., are structurally related to proteasome inhibitor epoxomicin, and show strong antiproteasomal activity. They exhibited strong cytotoxicity against lung adenocarcinoma (NCI-H460) and colon (HCT-116) cancer cell lines, as well as exquisite antiproliferative effects in the NCI-60 cell lines [32] (Table 1). Recently, carmaphycins conjugates with peptides and antibodies have been investigated in the design of novel antibody–drug conjugates (ADCs) for targeted delivery in cancer therapy [33].

### 2.9. Caylobolide

**Figure 10:**
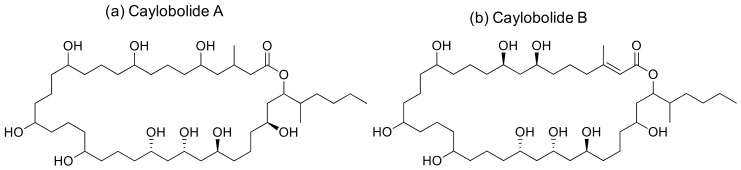
Chemical structure of caylobolides.

Caylobolide A and B are macrolactones (Figure 10) isolated from *Lyngbya majuscula* and *Phormidium* sp. that induce cytotoxicity against several cancer cell lines (Table 1). Caylobolide A exhibits cytotoxicity against the HCT-116 colon tumor with an IC_50_ of 9.9 µM [34], while the IC_50_ values of Caylobolide B against the HT29 colorectal adenocarcinoma and HeLa cervical carcinoma are 4.5 and 12.2 μM, respectively [35].

### 2.10. Coibamide A

**Figure 11:**
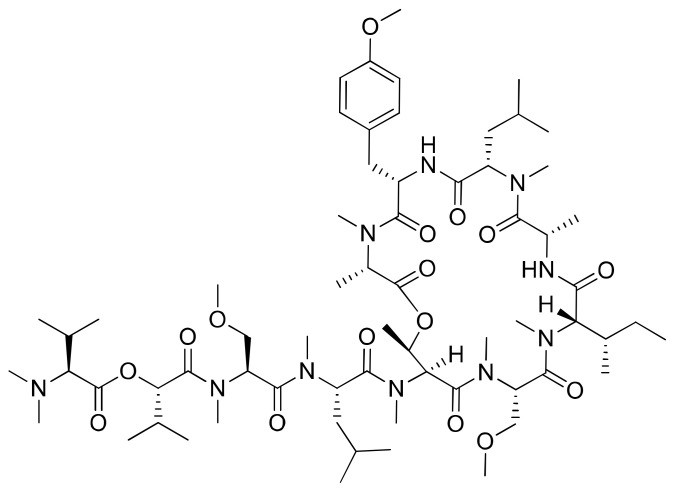
Chemical structure of coibamide A.

Coibamide A (Figure 11) is a novel N-methyl-stabilized antiproliferative depsipeptide extracted from *Leptolyngbya* sp. that induces concentration- and time-dependent cytotoxicity with a half-maximal effective concentration (EC_50_) < 100 nM in human SF-295 and U87-MG glioblastoma cells and mouse embryonic fibroblasts (MEFs) (Table 1). It induces mTOR-independent autophagy and cell death in human glioblastoma cells [36]. In addition, it displayed potent cytotoxicity towards the NCI-H460 lung cancer cells and mouse neuro-2a cells when used in nanomolar concentrations (LC_50_ < 23 nM). Previously, it was evaluated against an in vitro panel of 60 selective human cancer cell lines, including colon, breast and ovarian malignant tumors cells. The results indicated that coibamide A was found to be more effective against the MDA-MB-231 breast cancer cell line than against other cell lines [37]. Recently, an analog of coibamide A was synthesized that significantly inhibited tumor growth in vivo [38].

### 2.11. Cryptophycins 

**Figure 12:**
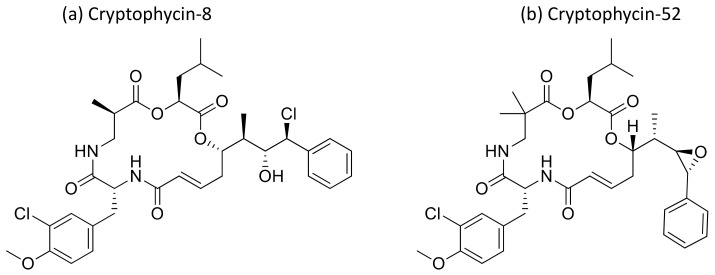
Chemical structure of cryptophicins.

Cryptophycin, a depsipeptide isolated from *Nostoc* sp. var. ATCC 53789 and GSV 224, is a potent anticancer agent. It inhibits microtubule assembly, and exhibited anti-tumorigenic activity against several solid tumors implanted in mice, including multidrug-resistant cancer cells (Table 1). The IC_50_ value was found to be less than 50 pM for multidrug-resistant cancer cell lines [39,40]. Several natural and synthetic analogs of cryptophycins (Figure 12) were reported and progressed to preclinical and clinical trials. Cryptophycin-8, a semi-synthetic analog, showed more efficient in vivo antiproliferative activity, leading it to act as an antitumor agent. Cryptophycin 52 (LY355703), isolated from *Nostoc* spp., induced cell cycle arrest in the G2/M phase and apoptosis via multiple pathways, such as both caspase-1- and caspase-3-dependent activation and Bcl-2 and Bcl-xL phosphorylation in several human prostate cancer cell lines [41]. A clinical phase II study of cryptophycin-52 revealed anticancer activity against advanced human non-small cell lung carcinoma (NSCLC) and platinum-resistant advanced ovarian cancer in patients [42], but failed due to neurotoxic side effects and limited in vivo efficacy [43]. Cryptophycins are promising drug candidates as their activity is not negatively affected by P-glycoprotein (a drug efflux system commonly found in multidrug-resistant tumor cell lines). Recently, cryptophycin conjugates with antibodies and peptides were developed for targeted drug delivery in cancer therapy [44]. The antiproliferative activities of RGD-cryptophycin and iso-DGR-cryptophycin conjugates were evaluated against human melanoma cells M21 and M21-L. They exhibit anticancer activity at nanomolar concentrations with different expression levels of integrin α_v_β_3_. In another study, the cryptophycin conjugate (RGD-cryptophycin) showed antiproliferative effects against M21 and M21-L human melanoma cell lines at nanomolar concentrations [45].

### 2.12. Curacin A

**Figure 13:**
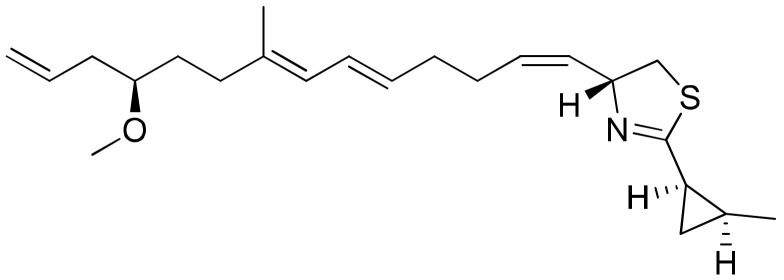
Chemical structure of curacin A.

Curacin A (Figure 13), an anti-tubulin agent isolated from *Lyngbya majuscula*, was screened against the NCI-60 panel of human tumor cell lines. It inhibits the binding of tubulin polymerization into the colchicine binding pocket, and arrests the cells in the G2/M phase of the cell cycle (Table 1). Due to its therapeutic potential as an antimitotic agent, curacin A was evaluated for clinical trials [46]. It exhibits cytotoxicity against renal, colon and breast cancer cell lines [47].

### 2.13. Desmethoxymajusculamide C 

**Figure 14:**
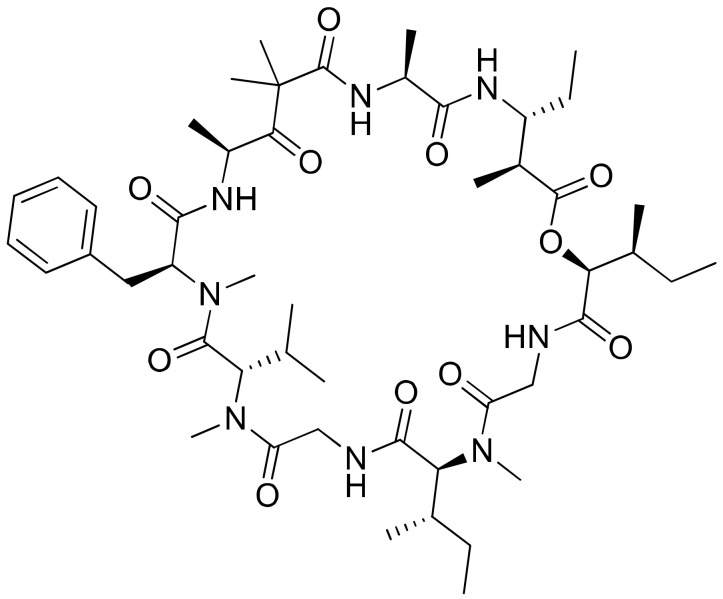
Chemical structure of desmethoxymajusculamide C.

Desmethoxymajusculamide C (Figure 14), a cyclic depsipeptide isolated from *L. majuscula*, exhibits strong antitumor potential against HCT-116 human colon carcinoma cells with an IC_50_ of 20 nM (Table 1). It helps in the destruction of the cell microfibrils networks by depolymerizing the actin cytoskeleton [48].

### 2.14. Dolastatins

**Figure 15:**
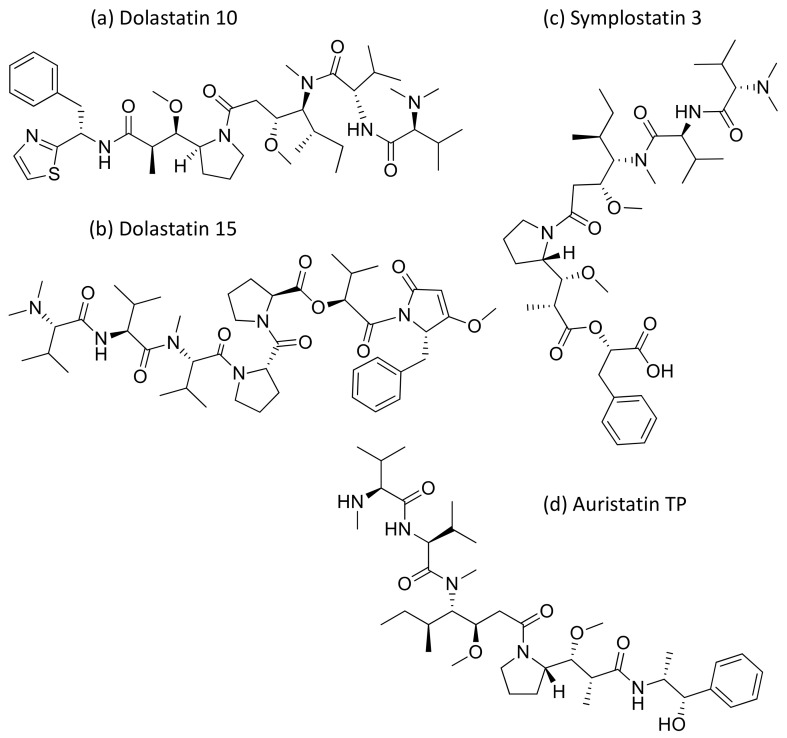
Chemical structure of some dolastatin analogs.

Dolastatins are peptides initially isolated from the sea hare *Dolabella auricularia* and later from marine cyanobacterial strains that induce cytotoxicity against cancer (Table 1). Dolastatins 10 and 15 (Figure 15a,b) are linear peptides isolated from a cyanobacteria, *Symploca* sp. VP642 [49]. They show cytotoxicity against several cancer cell lines due to their specific molecular interference with the dynamics of microtubule assembly, and induce an arrest in the G2/M phase of the cell cycle, leading to apoptosis. Several synthetic and semi-synthetic analogs of dolastatins 10 and 15 have been produced and are in clinical trials as anticancer drugs. Symplostatin 3 (Figure 15c), a dolastatin analog isolated from *Symploca* sp. VP452, disrupts microtubules, and showed IC_50_ values ranging from 3.9 to 10.3 nM against KB and LoVo cell lines, respectively [50]. Dolastatin 10 and its synthetic analog auristatin PE underwent early clinical trial phases, but have been withdrawn owing to a lack of efficacy and reports of peripheral neuropathy in patients. Nevertheless, a highly effective antibody–drug conjugate (ADC) named brentuximab vedotin, developed by Seattle Genetics, passed the clinical trial and was granted FDA approval in August 2011 for the treatment of Hodgkin’s lymphoma and anaplastic large cell lymphoma. Brentuximab vedotin is a combination of monomethyl auristatin E, linked with an antibody targeting CD30 on cancer cell surfaces [51]. Additionally, three more dolastatin 10 analogs were formulated as antibody–drug conjugate ADCs, namely, glembatumumab vedotin, SGN75 and ASG5ME, which are in the clinical trials [52]. The cyanobacterial peptide named glembatumumab vedotin has been approved for Phase II trials. It exhibited potent activity against breast and melanoma cancers with the maximum tolerant doses of 1.0–1.88 mg/kg [53]. Furthermore, a new analog, auristatin TP (Figure 15d), has been synthesized, which is a tyramide phosphate modification of dolastatin 10. It possesses improved anticancer properties owing to its increased bioavailability, with ED_50_ values ranging from <1.2 to 54.6 nM when tested against P388, NCI-H460 and MCF-7 cancer cell lines [54]. Besides this, dolastatin-15 derivative-based ADCs have also been developed that effectively kill human epidermal growth factor receptor (HER)2-positive cancer cells [55]. Recently, using trastuzumab (Herceptin^®^) as the antibody, Dol10-containing ADCs have been synthesized and investigated for in vitro cytotoxicity assays against HER2-positive (SK-BR-3) human tumor cells. Additionally, ADCs derived from Herceptin^®^ and PEG8-Dol10 effectively delayed tumor growth at a dose of 10 mg kg^−1^, and prolonged the survival time in mice bearing human ovarian SKOV-3 xenografts [56].

### 2.15. Grassypeptolides 

**Figure 16:**
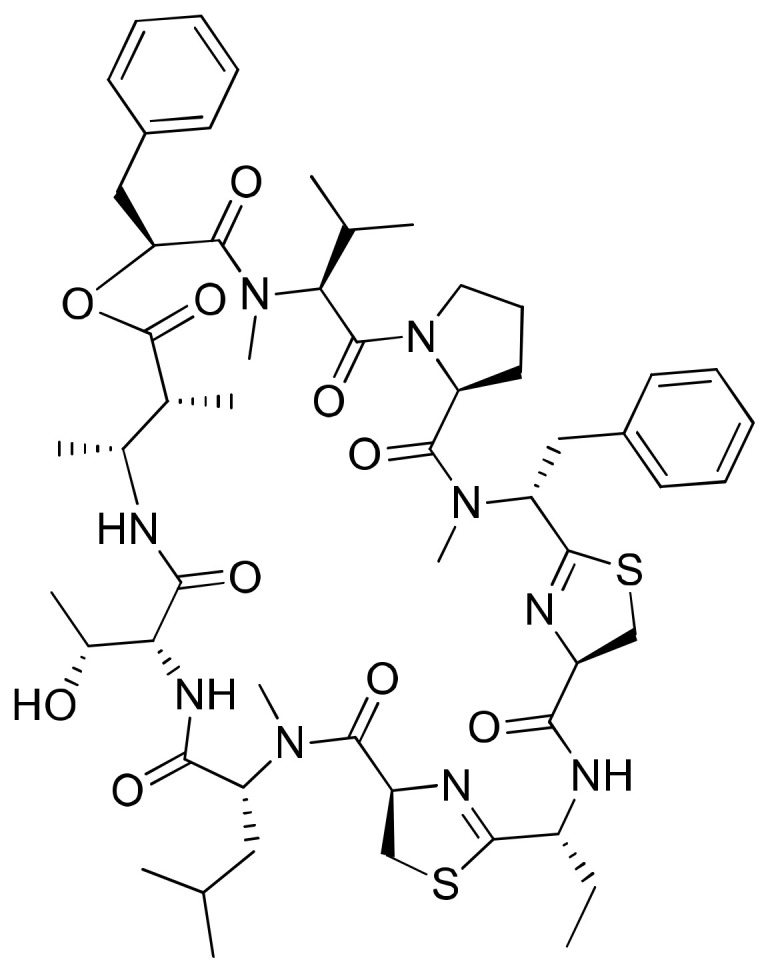
Chemical structure of grassypeptolide A.

Grassypeptolides A–C (Figure 16) are cyclic depsipeptides (containing bis-thiazoline) produced from *Lyngbya confervoides*, which reveal cytotoxicity against several cancer cells. The IC_50_ values for grassypeptolides A were evaluated against HT29 and HeLa cancer cell lines, and were found to be 1.22 and 1.01 µM, respectively (Table 1). Similarly, for grassypeptolides B and C, they were 4.97 and 2.93 µM and 76.7 and 44.6 nM, respectively. Grassypeptolide A and C both induce G1 cell cycle arrest at lower concentrations, but in HeLa cells they induce the G2/M cell-cycle arrest at higher concentrations [57]. Furthermore, grassypeptolide D and E reveal cytotoxicity against HeLa cells at IC_50_ values of 335 and 192 nM, respectively, and against mouse neuro-2a blastoma cells at IC_50_ values of 599 and 407 nM, respectively [58].

### 2.16. Hantupeptin A

**Figure 17:**
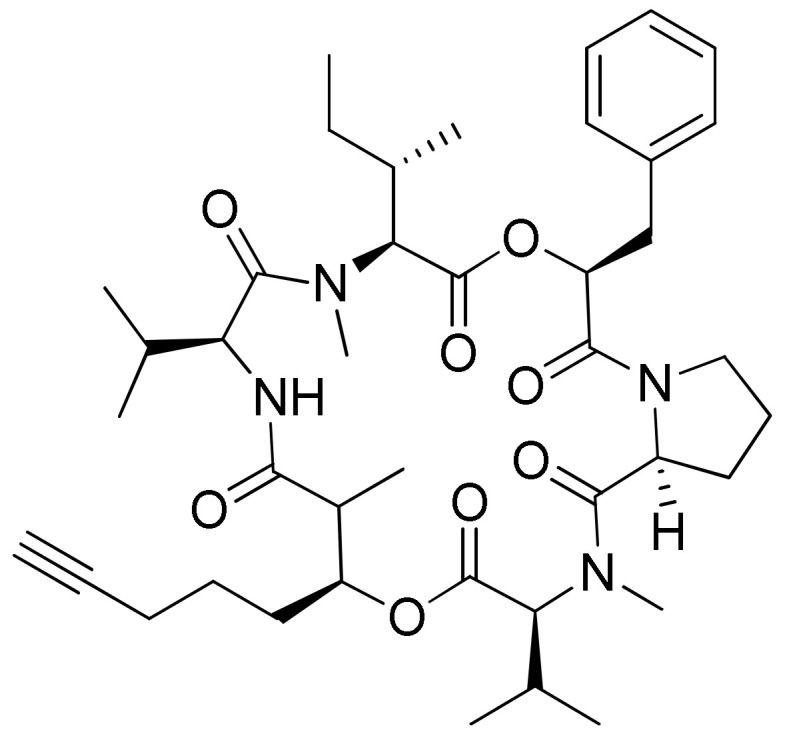
Chemical structure of hantupeptin A.

Hantupeptin A (Figure 17), a cyclodepsipeptide isolated from *L. majuscula,* exhibits cytotoxicity against the MOLT-4 leukemia and MCF-7 breast cancer cell line with IC_50_ values ranging from 32 nM to 4.0 μM, respectively [59] (Table 1).

### 2.17. Hectochlorin

**Figure 18:**
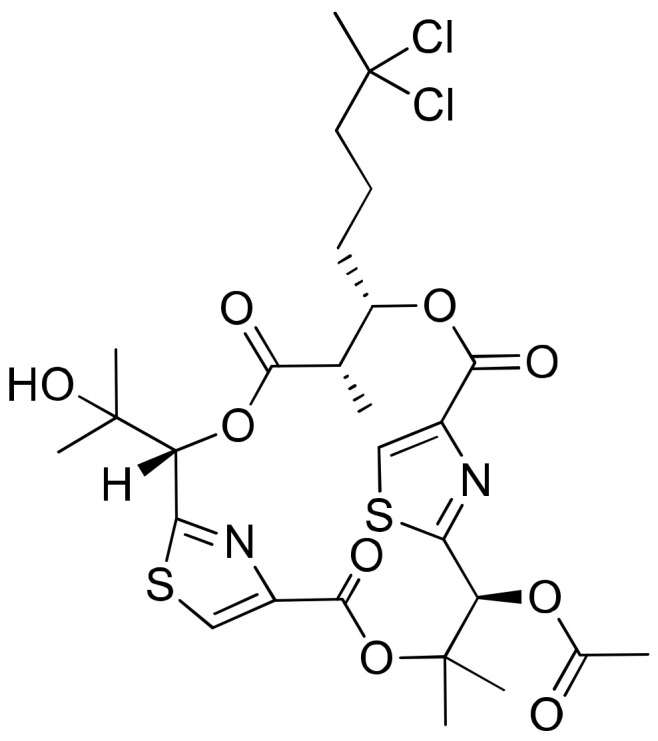
Chemical structure of hectochlorin.

Hectochlorin (Figure 18) was isolated from *L. majuscula*, and promotes actin polymerization with an EC_50_ value of 20 μM in human non-tumoral kidney cells PtK2. It also displays cytotoxicity against the NCI-60 panel of cancer cell lines (Table 1). Among these, 23 cancer cell lines, such as colon melanoma, renal cells and ovarian cells, showed the strongest cytotoxicity. The IC_50_ values range from 20 to 300 nM for CA46 human Burkitt lymphoma cell and PtK2 cells, respectively. However, the dose–response curve of hectochlorin was flat, suggesting that the compound is more antiproliferative than cytotoxic [60].

### 2.18. Hierridin B

**Figure 19:**
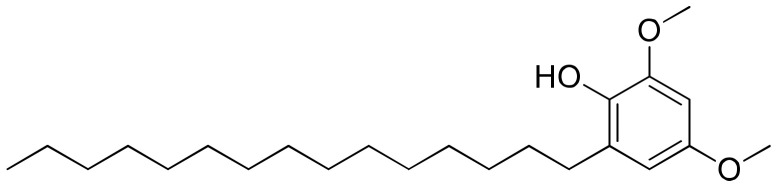
Chemical structure of hierridin B.

Hierridin B (Figure 19), a polyketide isolated from the *Cyanobium* sp. LEGE 06113, was tested in a panel of eight human cancer cell lines, and showed selective cytotoxicity only against the colon adenocarcinoma cell line HT-29, with an IC_50_ value of 0.1 mM [60] (Table 1).

### 2.19. Hoiamide D

**Figure 20:**
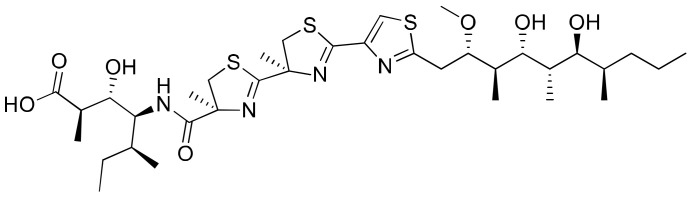
Chemical structure of hoiamide D.

Hoiamide D, isolated from *Symploca* sp, consists of a unique triheterocylic system structurally comprising two consecutive thiazolines and a thiazole, as well as an unusual isoleucine moiety (Figure 20). The carboxylate anion of hoiamide D was reported to inhibit p53/HDM2 (human homolog of MDM2) interaction with an EC_50_ of 4.5 mM (Table 1). Besides this, it also possesses significant sodium channel-activating properties. [61].

### 2.20. Hormothamnin A 

**Figure 21:**
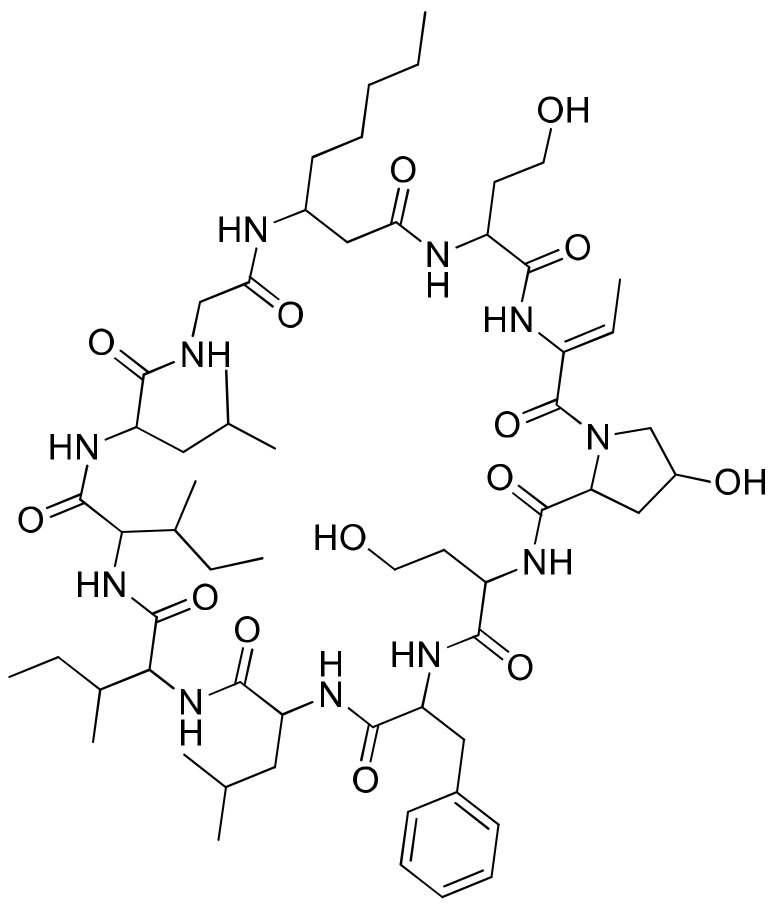
Chemical structure of hormothamnin A.

Hormothamnin A (Figure 21), a cyclic undecapeptide (containing six common and five uncommon amino acid residues) isolated from *Hormothamnion enteromorphoides*, exhibits significant cytotoxicity against several solid cancer cell lines, such as human colon cells (HCT-116), human lung cells (SW1271 and A529) and murine melanoma cells (B16-F10), with IC_50_ values ranging from 0.13 to 0.72 µg/mL [62] (Table 1).

### 2.21. Itralamides A and B

**Figure 22:**
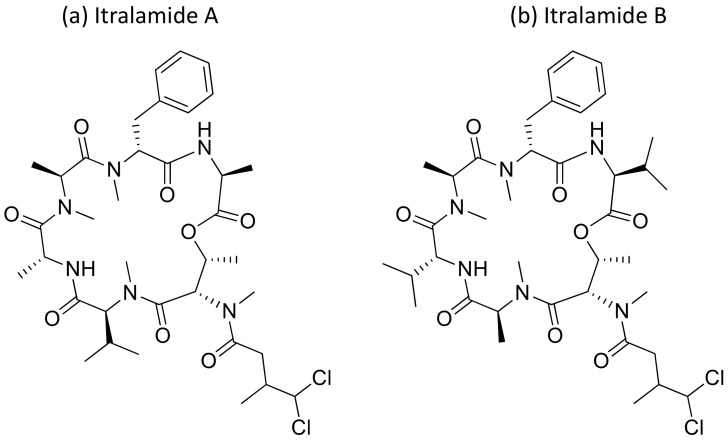
Chemical structure of itralamides A and B.

Itralamides A and B (Figure 22), cyclodepsipeptides isolated from *L. majuscula*, exhibit cytotoxicity against human embryonic kidney cells 293 (HEK293). Itralamide A showed lower cytotoxicity than Itralamide B against HEK293 cells with an IC_50_ of 6 µM [40,63] (Table 1).

### 2.22. Lagunamides

**Figure 23:**
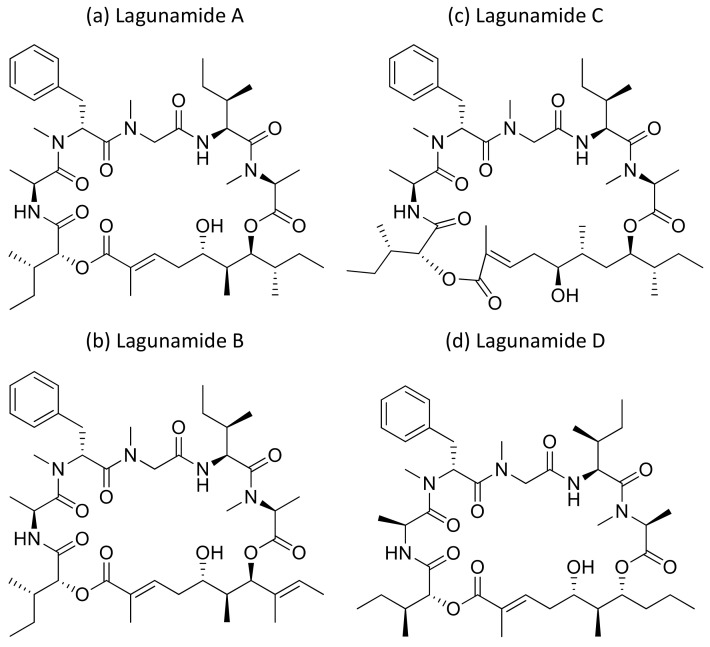
Chemical structure of lagunamides.

Lagunamide A (Figure 23a), isolated from *L. majuscula*, showed cytotoxicity against a panel of cancer cell lines, such as HCT8 (human colorectal cancer), P388 (murine leukemia), PC3 (human prostate cancer), A549 (human lung cancer) and SK-OV3 (human ovarian cancer), with IC_50_ values ranging from 1.6 nM to 6.4 nM (Table 1). The molecule exhibits an antiproliferative effect via mitochondria-mediated apoptosis against HCT8 and MCF7 (breast) cancer cell lines [64]. Furthermore, lagunamide A and B (Figure 23a,b) exhibit cytotoxicity against P388 murine leukemia cell lines with IC_50_ values of 6.4–20.5 nM, respectively [65]. In another study, Lagunamide C (Figure 23c) revealed strong cytotoxicity against A549, PC3, P388, HCT8, and SK-OV3 cell lines with IC_50_ values ranging from 2.1 to 24.4 nM [66]. In addition, Lagunamide A and D (Figure 23a,d) exhibited antiproliferative activity against A549 human lung adenocarcinoma cells with an IC_50_ value of 6.7–7.1 nM, respectively [67]. Recently, a molecular mechanism study in A549 human lung adenocarcinoma cells showed that lagunamide-A induced caspase-mediated mitochondrial apoptosis [68].

### 2.23. Largazole

**Figure 24:**
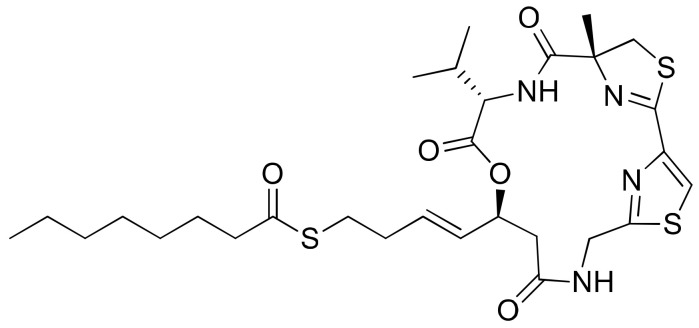
Chemical structure of largazole.

Largazole (Figure 24), a cyclic depsipeptide isolated from *Symploca* sp., is a highly potent class I histone deacetylase (HDAC) inhibitor, and is currently being used for the development of anticancer drugs. It has been screened to evaluate its cytotoxicity against many epithelial and fibroblastic cancer cell lines [69] (Table 1). Generally, largazole acts as a prodrug, and upon hydrolysis of the thioester, a largazole thiol releases that function as a reactive molecule. The resultant molecule then complexes with Zn^2+^ and inhibits class I histone deacetylase. Beside its HDAC inhibitory property, largazole exhibits a wider range of in vitro and in vivo biological activities, for example antitumor, antiosteogenic and antifibrotic activities [70,71,72]. When largazole was used together with dexamethasone (a synthetic glucocorticoid steroid), it induced E-cadherin localization at the plasma membrane in triple-negative breast cancers and suppressed cancer invasion [73]. In another study, largazole sensitized Epstein–Barr virus-positive (EBV^+^) lymphoma cells to ganciclovir (an antiherpes viral drug) at nanomolar concentrations [74]. Recently, largazole and its analogs were found to inhibit a ubiquitin-activating enzyme (E1) during ubiquitination [75].

### 2.24. Laxaphycins

**Figure 25:**
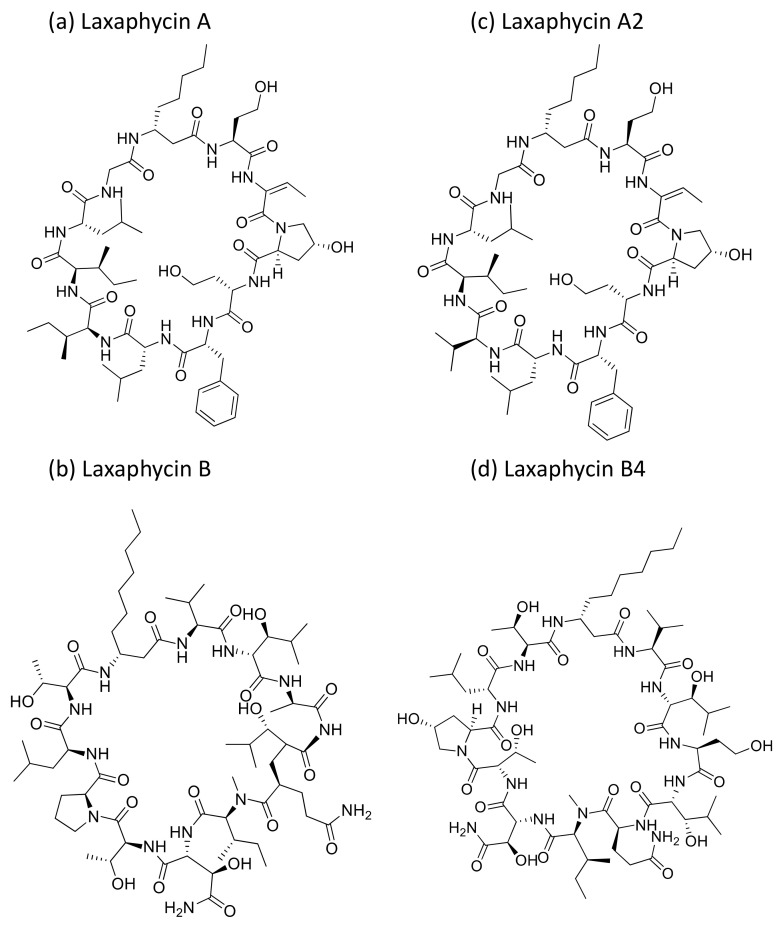
Chemical structure of laxaphycins.

Laxaphycin A and B (Figure 25a,b) are cyclic peptides generally isolated from *Anabaena laxa*. Laxaphycin A showed weak cytotoxicity against certain cancer cell lines (Table 1) such as A549, MCF7, PA1 (ovarian teratocarcinoma), PC3, DLD1 (colorectal adenocarcinoma) and M4Beu (melanoma). However, laxaphycin B revealed strong anticancer activity against both resistant and sensitive cancer cell lines [76]. Recently, laxaphycins B4 and A2 (Figure 25c,d) were isolated from *H. enteromorphoides*, along with the known compound laxaphycin A. Laxaphycin B4 exhibited antiproliferation activity against human colon cancer HCT116 cell lines with an IC_50_ value of 1.7 µM, whereas laxaphycins A and A2 revealed weak activities [77].

### 2.25. Lyngbyabellins

**Figure 26:**
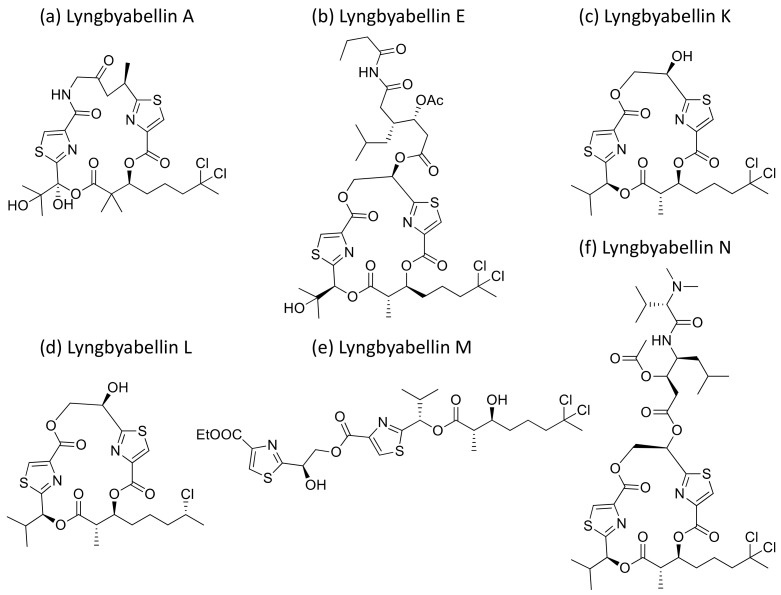
Chemical structure of lyngbyabellins.

Lyngbyabellin A and E (Figure 26a,b), isolated from *L. majuscula*, are hectochlorin-related lipopeptides. They possess potent actin polymerization activity. Lyngbyabellin A exhibited moderate cytotoxicity against human cervical (KB) and colon (LoVo) cancer cell lines with IC_50_ values of 0.03 µg/mL and 0.5 µg/mL, respectively (Table 1) [78]. In vivo trials suggested that lyngbyabellin A is toxic to mice. It disrupts the cellular microfilament network in A-10 cells at 0.01–5.0 μg/mL concentrations [78]. When compared to the above, Lyngbyabellin B was found to be slightly more cytotoxic in in vitro trials [79]. Furthermore, Lyngbyabellin E showed cytotoxicity against human lung tumors (NCI-H460) and neuro-2a mouse neuroblastoma cell lines with LC_50_ values between 0.2 and 4.8 µM [80]. Lyngbyabellin N (Figure 26f) exhibited strong cytotoxic activity against the HCT116 colon cancer cell line (IC_50_ = 40.9 ± 3.3 nM), while lyngbyabellins K, L, M and 7-epi-lyngbyabellin L (Figure 26c–e) do not show any activity [81].

### 2.26. Lyngbyastatins

**Figure 27:**
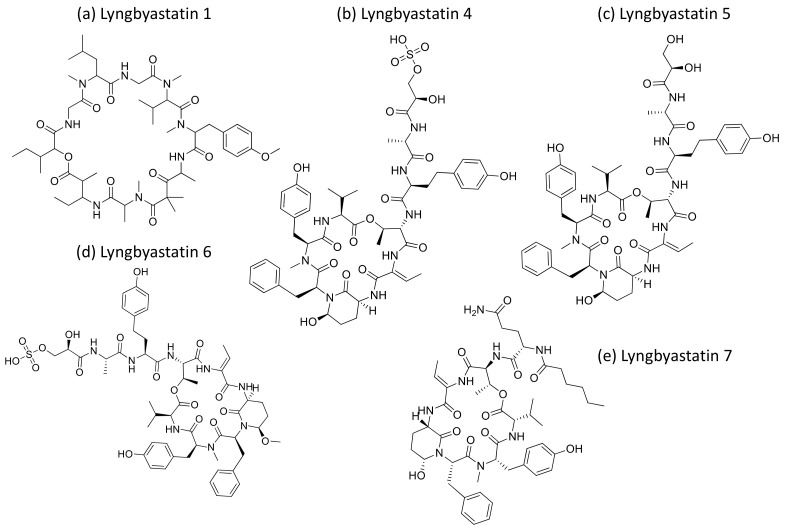
Chemical structure of lyngbyastatins.

Lyngbyastatins (Figure 27) are cyclic depsipeptides isolated from *Lyngbya* sp. that exhibit strong serine proteases inhibition. Lyngbyastatins 4 to 7 are potent inhibitors of elastase, with IC_50_ values ranging from 120 to 210 nM [82]. Furthermore, Lyngbyastatins 8–10, isolated from *Lyngbya semiplena*, inhibit porcine pancreatic elastase, with IC_50_ values ranging from 120 to 210 nM [83] (Table 1).

### 2.27. Malyngamides 

**Figure 28:**
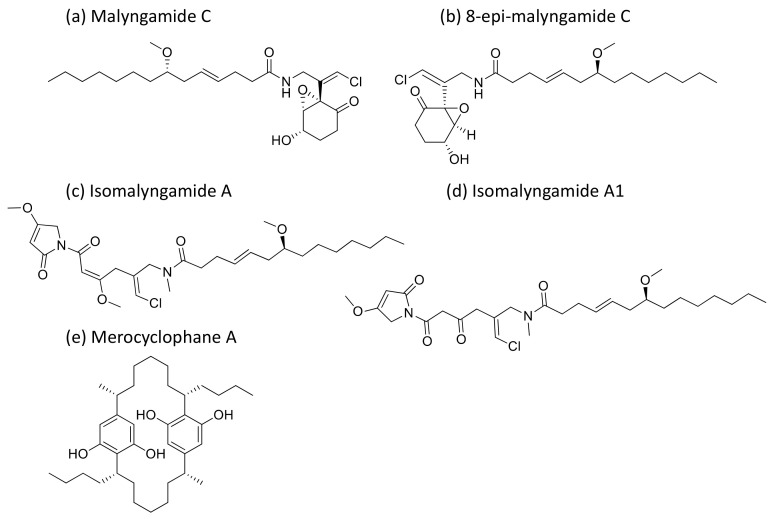
Chemical structure of malyngamides and derivatives.

Malyngamides (Figure 28), small amides produced by *L. majuscula*, exhibit antiproliferative activity against tumor cells at the level of nanomolar to micromolar concentrations (Table 1). Isomalyngamides A and A-1, analogs of malyngamide, inhibit tumor proliferation by inactivating the expression and phosphorylation of FAK and Akt through the β1 integrin-mediated antimetastatic pathway [84]. Another analog, malyngamide C and 8-epi-malyngamide C (Figure 28b), showed cytotoxicity against HT29 colon cancer cells with IC_50_ values of 5.2 and 15.4 µM, respectively [85]. Additionally, Merocyclophanes A (Figure 28e) and B, isolated from *Nostoc* sp. (UIC 10022A), exhibited antiproliferative activity against the above cell lines with IC_50_ values of 3.3 and 1.7 µM, respectively [86]. Recently, three new malyngamides were isolated from *Moorea producens* and, in particular, 6,8-di-O-acetylmalyngamide-2 showed strong anticancer activity by activating adenosine monophosphate-activated protein kinase (AMPK) [87].

### 2.28. Nocuolin A 

**Figure 29:**
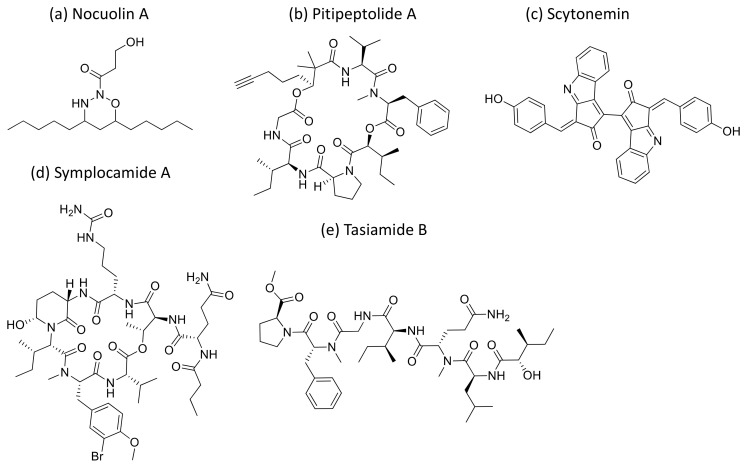
Chemical structure of nocuolin A (**a**), pitipeptolide A (**b**), scytonemin (**c**), symplocamide A (**d**) and Tasiamide (**e**).

Nocuolin A (Figure 29a), a natural oxadiazine isolated from some of the species belonging to *Nostoc*, *Nodularia* and *Anabaena* genera, induces cell death and exhibits anti-proliferative activity against various human cancer cells, specifically against p53-mutated cell lines with IC_50_ values ranging from 0.7 to 4.5 µM [88] (Table 1).

### 2.29. Pitipeptolides 

Pitipeptolides (Figure 29b) are cyclic depsipeptides isolated from *L. majuscula*. Pitipeptolides A, B, and C–F revealed cytotoxicity against HT 29 colon adenocarcinoma and MCF-7 breast cancer cell lines, with an IC_50_ value between 10 and 100 µM [89] (Table 1).

### 2.30. Scytonemin

Scytonemin (Figure 29c), an aromatic indole alkaloid extracted from *Stigonema* sp., showed antiproliferative activity against several human fibroblast and endothelial cell lines. It inhibits the human polo-like kinase activity that plays a crucial role in the regulation of the cell cycle at the G2/M transition [90] (Table 1).

### 2.31. Symplocamide A 

Symplocamide A (Figure 29d), a cyclodepsipeptide isolated from *Symploca* sp., showed potent cytotoxicity against non-small cell lung cancer cells NCI-H460 (IC_50_ = 40 nM) and neuro-2a neuroblastoma cells (IC_50_ = 29 nM) by inhibiting serine proteases activity (IC_50_ = 0.38 µM) [91] (Table 1).

### 2.32. Tasiamides

Tasiamides are linear peptides isolated from *Symploca* sp. and tasiamide B (Figure 29e) that showed strong cytotoxicity against human cervical adenocarcinoma (KB) cells, with an IC_50_ value of 0.8 µM [92]. In another study, eighteen analogs of tasiamide were screened against KB cells and human non-small cell lung tumor A549 cells, with IC_50_ values between 1.29 and 12.88 μM, respectively [93] (Table 1).

### 2.33. Veraguamides

**Figure 30:**
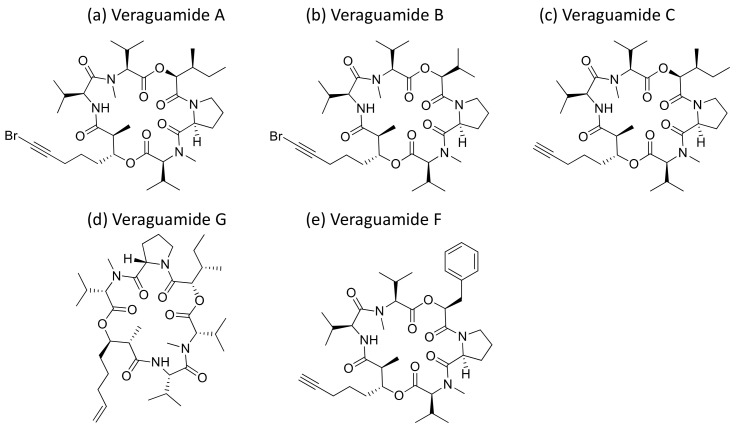
Chemical structure of veraguamides.

Veraguamides A–C and H–L cyclodepsipeptides (Figure 30) were isolated from *Oscillatoria margaritifera*, and veraguamides A–G were also isolated from *Symploca hydnoides*. All showed a cytotoxic effect (Table 1). Veraguamide A was found to be highly toxic against the human lung cancer cell line NCI-H460 (LD_50_ = 141 nM) [94]. Moreover, veraguamides A–G exhibited moderate to weak cytotoxicity against both the HT 29 and HeLa cell lines [95].

### 2.34. Miscellaneous

**Figure 31:**
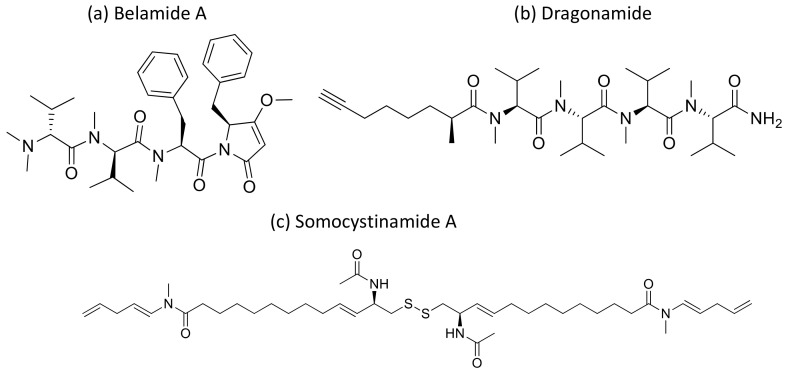
Chemical structure of belamide A (**a**), dragonamide (**b**) and somocystinamide A (**c**).

Kempopeptins A and B (cyclodepsipeptides from *Lyngbya* sp.), Bouillomides A and B (depsipeptides from *Lyngbya bouillonii*), Molassamide (depsipeptide from *Dichothrixutahensis*), Largamides (cyclic peptides from *Lyngbya confervoides*), Pompanopeptin A, (cyclic peptide from *L. confervoides*), and all dolastatin 13 analogs, strongly inhibit non-caspase proteases’ activity with IC_50_ values ranging from 0.32 to 25.0 μM [4]. Kempopeptin C has recently been isolated as a serine protease inhibitor. It is a chlorinated analog of kempopeptin B that inhibited the migration of invasive breast cancer (MDA-MB-231) cells by 37–60% at the 10 and 20 µM concentrations [96]. Furthermore, Belamide A (Figure 31a), a linear tetrapeptide isolated from *Symploca* sp., exhibits cytotoxicity against HCT-116 colon cancer with an IC_50_ of 0.74 µM by disturbing microtubule formation [97] (Table 1). 

Moreover, dragonamide (Figure 31b), a lipopeptide isolated from *L. majuscula*, induces cytotoxicity on P-388, A-549, HT-29 and MEL-28 cancer cells with an IC_50_ > 1 μg/mL [98] (Table 1). Recently, a cyanobacterial species named *Fischerella* has been isolated from the Nile river. The different compounds isolated from it have been explored and were found to be active against breast cancer (MCF-7), liver cancer (HepG-2), colon cancer (HCT116), and lung cancer (A549) cell lines. However, its crude extract BS1-EG exhibited various effects on all tested cell lines [99].

**Figure 32:**
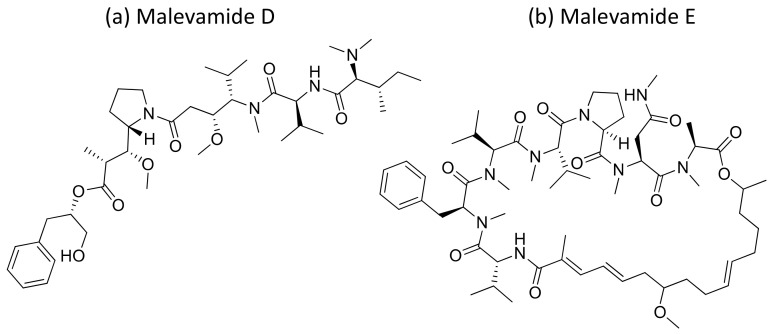
Chemical structure of mavelamide D and E.

**Figure 33:**
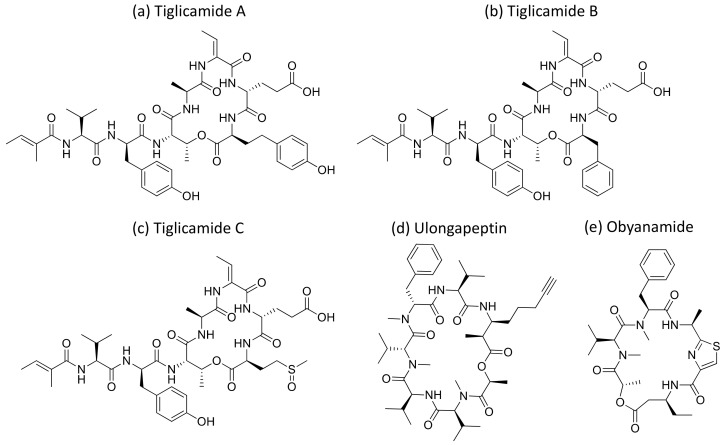
Chemical structure of tiglicamide A to C (**a**–**c**), ulongapeptin (**d**) and obyanamide (**e**).

Malevamide D and E (Figure 32) are peptide esters isolated from *S. hydnoides* that induce cytotoxicity against P-388, A-549, HT-29, and SK-MEL-28 (human melanoma) cell lines with IC_50_ values ranging from 0.3 to 0.7 nM [100] (Table 1). Somocystinamide A (Figure 31c), a lipopeptide isolated from *L. majuscula,* exhibits cytotoxicity on Jurkat (IC_50_ 3nM) and CEM leukemia (IC_50_ 14 nM), A549 lung carcinoma (IC_50_ 46 nM), Molt4 T leukemia (IC_50_ 60 nM), M21 melanoma (IC_50_ 1.3 nM) and U266 myeloma (IC_50_ 5.8 µM) cell lines by inducing apoptosis via caspase 8 [101] (Table 1). Tiglicamides A–C (Figure 33a–c), Ulongapeptin (Figure 33d) and Obyanamide (Figure 33e), which are cyclic depsipeptides isolated from *Lyngbya* sp., induce cytotoxicity in cancer cell lines, such as KB cells, with IC_50_ values ranging from 2.14 to 7.28 µM, 0.63 µM and 0.58 µg/mL, respectively, and later inhibit the serine proteases pathway [102,103,104] (Table 1). Wewakpeptins, a depsipeptide from *L. semiplena*, showed anticancerous potential against NCI-H460 lung cancer with an LD_50_ of 0.4 µM [105] (Table 1).

**Figure 34:**
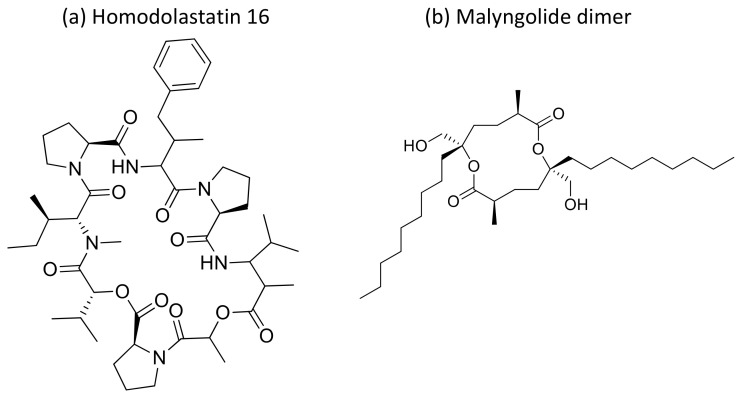
Chemical structure of homodolastatin 16 (**a**) and malyngolide dimer (**b**).

**Figure 35:**
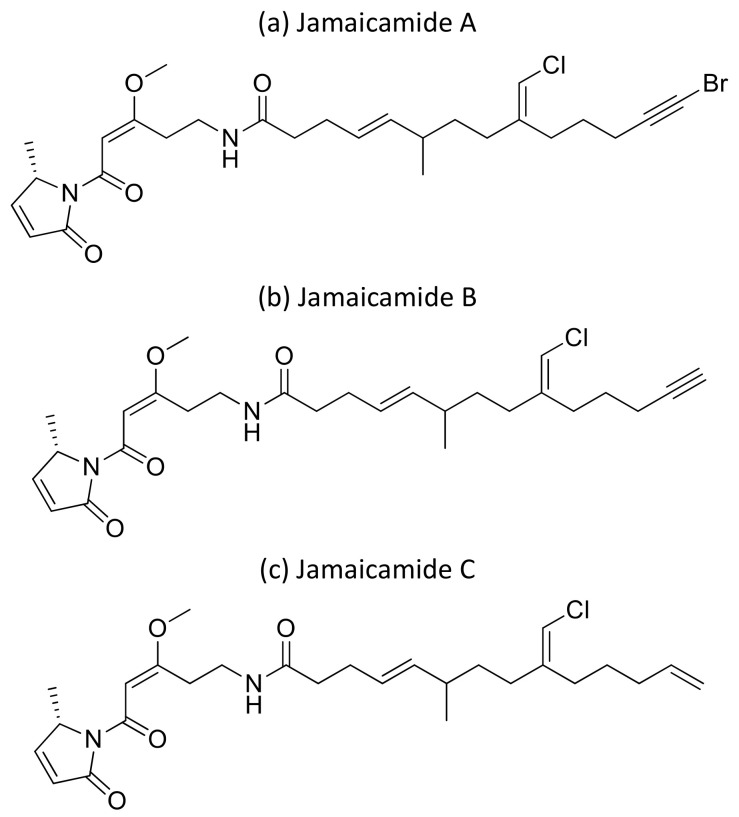
Chemical structure of jamaicamides.

Moreover, the cyclic depsipeptide Homodolastatin 16 (Figure 34a) [106], the polyketide-peptides Jamaicamide A–C (Figure 35) [107], and the cyclodepside malyngolide dimer (Figure 34b) [108], all isolated from *L. majuscula*, induce cytotoxicity against several human tumor cells such as the oesophageal WHCO1 and WHCO6, cervical ME180, breast MCF-7 and MDA-MB-231, and lung NCI-H460 cancer cell lines (Table 1). Recent evidence has shown the antiproliferative activity of the depsipeptides anaenamide A and B extracted from Guam cyanobacteria [109]. Finally, Tutuilamides A–C, which are vinyl-chloride-containing cyclodepsipeptides identified from cyanobacterial field collections from American Samoa and Palmyra Atoll, display potent elastase inhibitory activity, together with moderate antiproliferative activity toward H-460 lung cancer cells [110].

**Table 1:** Summarized data of cyanobacterial secondary metabolites with anti-cancerous potential.

Compound Name	Analogs	Specific Class/Type	Source	In Vitro*/*In Vivo Cancer Cell Lines Screened	IC_50_/LC_50_/ED_50_/GI_50_ Values	Molecular Targets	References
**Ankaraholide A**		Glycosylated swinholide	*Geitlerinema*	NCI-H460; MDA-MB-435	IC_50_ = 119 nM IC_50_ = 8.9 nM	Loss of filamentous actin	[8]
**Apratoxin**	Apratoxin A Apratoxin B–D Apratoxins A and E hybrid Oxazoline Apratoxin S10	Cyclic depsipeptide	*Lyngbya* sp.	LoVo, KB; HT29, HeLa, U2OS; NCI-H460	IC_50_ = 0.36 nM IC_50_ = 0.52 nM IC_50_ = 2.6 nM	Cell cycle arrest, apoptosis, secretory pathway inhibition	[9,10,11,12,13,14,15,16]
**Aurilide**	Aurilide B Aurilide C	Cyclic depsipeptide	*Dolabellaauriculari* and *Lyngbya majuscula*	NCI-H460, Neuro2a ; NCI-60 panel	LC_50_ = 0.01 to 0.13 µM GI_50_ <10 nM	Mitochondrial induced apoptosis	[17]
**Belamide A**		Linear tetrapeptide	*Symploca* sp	HCT-116	IC_50_ = 0.74 µM	Microtubule disruption	[97]
**Bisebromoamide**		Peptide	*Lyngbya* sp	HeLa S3; 769-P; 786-O;	IC_50_ = 0.04 µg/mL IC_50_ = 1.63µM IC_50_ = 2.11µM	Actin filaments stabilization, protein kinase inhibition, induces apoptosis through ERK and mTOR inhibitions	[19,20,21,22,23]
**Biselyngbyaside**	Biselyngbyaside A Biselyngbyolide B, C, E	Glicomacrolide	*Lyngbya* sp.	HeLa S3, HeLa, HL-60	IC_50_ = 0.1 μg/ml	Induces ER stress and apoptosis	[24,25]
**Borophycin**			*Nostoc linckia*, *N. spongiaeforme*	LoVo, KB			[26]
**Calothrixin A**	Calothrixin A Calothrixin B Isothiacalothrixin B	Pentacyclic indolophenanthridine	*Calothrix*	HeLa, HCT-116; NCI-H460	IC_50_ = 0.32 µM; GI_50_ = 1 nM	Induced apoptosis and cell cycle arrest in S and G_2_/M phase	[27,28,29,30,31]
**Carmaphycin**	Carmaphycins A and B		*Symploca* sp.	NCI-H460, HCT-116		Proteasome inhibitor	[32]
**Caylobolide**	Caylobolide A and B	Macrolactone	*Lyngbya majuscula*, *Phormidium* spp	HCT-116; HT29; HeLa	IC_50_ = 9.9 µM IC_50_ = 4.5 µM IC_50_ = 12.2 μM		[34,35]
**Coibamide A**		Cyclic depsipeptide	*Leptolyngbya* sp.	SF-295, U87-MG, MEFs; NCI-H460, neuro-2a, MDA-MB-231	EC_50_ <100 nM LC_50_< 23 nM	Induces mTOR-independent autophagy and cell death	[36,37]
**Cryptophycin**	Cryptophycin-8 Cryptophycin 52 (LY355703) RGD-cryptophycin and *iso*DGR-cryptophycin conjugates	Cyclic depsipeptide	*Nostoc* sp. var. ATCC 53789 and GSV 224	NSCLC, multidrug-resistant cancer cell lines (M21, M21-L), platinum-resistant advanced ovarian cancer	IC_50_ > 50 pM	Inhibits microtubule assembly, induces cell cycle arrest in G_2_/M phase and apoptosis	[39,40,41,42]
**Curacin A**		Lipopeptide	*Lyngbya majuscula*	NCI-60 panel		Tubulin polymerization inhibition, cell cycle arrest at G_2_/M phase	[46,47]
**Desmethoxymajusculamide C**		Cyclic depsipeptide	*L. majuscula*	HCT-116	IC_50_= 20 nM	Destroys cell microfibrils networks	[48]
**Dolastatins**	Dolastatins 10 Dolastatins 15 Symplostatin 3 Auristatin TP	Linear Pentapeptide	*Dolabella auricularia Symploca* sp. VP642 *Symploca* sp. VP452	KB, LoVo; P388, NCI-H460, MCF-7,	IC_50_ = 3.9 nM IC_50_= 10.3 nM ED_50_ < 1.2 to 54.6 nM	Interferes with the dynamics of microtubule assembly and induces an arrest in the G_2_/M phase of the cell cycle, leading to apoptosis	[49,50,54]
**Dragonamide**		Lipopeptide	*Lyngbya majuscula*	P-388, A-549, HT-29, MEL-28	IC_50_ > 1 μg/ml		[98]
**Grassystatins**	Grassypeptolides A-E	Cyclic depsipeptides	*Lyngbya confervoides*	HT29; HeLa; neuro-2a	IC_50_= 76.7 nM to 4.97 µM IC_50_ = 44.6 nM to 2.93 µM; IC_50_ = 0.41 to 0.60 µM	Induces G_1_ and G_2_/M phase cell cycle arrest	[57,58]
**Hantupeptin A**		Cyclodepsipeptide	*Lyngbya majuscula*	MOLT-4; MCF-7	IC_50_ = 32 µM IC_50_ = 4.0 μM		[59]
**Hectochlorin**		Lipopeptide	*Lyngbya majuscula*	CA46, PtK2; NCI-60 panel	IC_50_ = 20 nM IC_50_ = 0.3 µM GI_50_ = 5.1 µM	Cell cycle inhibition by promoting actin polymerization	[60]
**Hierridin B**		Polyketide	*Cyanobium* sp.	HT-29	IC_50_ = 0.1 mM		[61]
**Hoiamide**	Hoiamide D	Cyclic depsipeptide	*Symploca* sp.		EC_50_ = 4.5 mM	Inhibits p53/HDM2 and activates sodium-channels	[62]
**Homodolastatin 16**		Cyclic depsipeptide	*Lyngbya majuscula*	WHCO1; WHCO6; ME180	IC_50_ = 4.3 µg/mL IC_50_ = 10.1 µg/mL; IC_50_ = 8.3 µg/ml		[106]
**Hormothamnin A**		Cyclic undecapeptide	*Hormothamnion enteromorphoides*	HCT-116, SW1271, A529, B16-F10	IC_50_ = 0.13 to 0.72 µg/ml		[40]
**Itralamide**	Itralamide A and B	Cyclodepsipeptides	*L. majuscula*	HEK293	IC_50_ = 6 µM		[63]
**Jamaicamides A–C**		Polyketide-Peptides	*Lyngbya majuscula*	NCI-H460	LC_50_ ~ 15 µM		[107]
**Lagunamide**	Lagunamides A Lagunamides B Lagunamide C Lagunamide D	Cyclic depsipeptide	*Lyngbya majuscula*	HCT8, P388, PC3, SK-OV3, MCF7; A549	IC_50_ = 1.6 nM to 24.4 nM; 6.7 to 7.1 nM	Caspase-mediated mitochondrial apoptosis	[64,65,66,67,68]
**Largazole**		Cyclic depsipeptide	*Symploca* sp.	Epithelial and fibroblastic cancer cell lines		Inhibit class I histone deacetylase	[69]
**Laxaphycin**	Laxaphycin A and B Laxaphycins B4 and A2	Cyclic peptides	*Anabaena laxa.* *Hormothamnion enteromorphoides*	HCT-116; A549, MCF7, PA1, PC3, DLD1, M4Beu,	IC_50_ = 1.7 µM		[76,77]
**Lyngbyabellin**	Lyngbyabellin A and E Lyngbyabellin K-N	Cyclic depsipeptide	*L. majuscula*	KB, LoVo; HCT116; NCI-H460, neuro-2a	IC_50_ = 0.03 µg/mL IC_50_ = 0.5 µg/mL; IC_50_ = 40.9 nM LC_50_ = 0.2 to 4.8 µM	Possesses actin polymerization activity, disrupts the cellular microfilament network	[78,79,80,81]
**Lyngbyastatin**	Lyngbyastatins 1 Lyngbyastatins 4–7	Cyclic depsipeptide	*Lyngbya majuscula* *Lyngbya semiplena*	None: in vitro enzyme assay	IC_50_ = 120 to 210 nM	Inhibits serine proteases (elastase)	[82,83]
**Malevamide**	Malevamide D Malevamide E	Peptide ester	*Symploca hydnoides*	P-388, A-549, HT-29, MEL-28	IC_50_ = 0.3 to 0.7 nM		[100]
**Malyngamide**	Isomalyngamides A and A-1 malyngamide C and 8-epi-malyngamide C Merocyclophanes A and B 6,8-di-O-acetylmalyngamide 2 Malyngamide C, J and K	Fatty acid amine	*Lyngbya sp.* Nostoc sp. (UIC 10022A), *Moorea producens*	MCF-7, MDA-MB-231 HT29	IC_50_ = 4.6 µM IC_50_ = 2.8 µM IC_50_ = 1.7 to 15.4 µM	Inactivates the expression of p-FAK, FAK, p-Akt and Akt through β1 integrin-mediated antimetastatic pathway and activates AMPK	[84,85,86,87]
**Malyngolide dimer**		Cyclodepside	*Lyngbya majuscula*	NCI-H460	IC_50_ ~ 19 µM		[108]
**Nocuolin A (NoA)**		Oxadiazine	*Nostoc, Nodularia and Anabaena*	p53 mutated cell lines	IC_50_ = 0.7 to 4.5 µM	Induces cell death and exhibits anti-proliferative activity	[88]
**Obyanamide**		Cyclic depsipeptide	*Lyngbya confervoides*	KB	IC_50_ = 0.58 µg/ml		[104]
**Pitipeptolides**	Pitipeptolides A-B and C-F	Cyclic depsipeptides	*Lyngbya majuscula*	HT 29, MCF-7	IC_50_ = 10 to 100 µM		[89]
**Scytonemin**		Polysaccharide	*Stigonema* sp	Human fibroblast and endothelial cell lines		Inhibits human polo-like kinase activity that plays a crucial role in the regulation of the cell cycle at the G_2_/M transition	[90]
**Somocystinamide A**		Lipopeptide	*Lyngbya majuscula*	Jurkat; CEM; A549; Molt4 T; M21; U266	IC_50_ = 3 nM IC_50_ = 14 nM IC_50_ = 46 nM IC_50_ = 60 nM IC_50_ = 1.3 µM IC_50_ = 5.8 µM	Induces apoptosis selectively via Caspase 8	[79,101]
**Symplocamide**	Symplocamide A	Cyclodepsipeptide	*Symploca* sp.	NCI-H460; neuro-2a	IC_50_ = 40 nM IC_50_ = 29 nM	Serine proteases inhibitor	[91]
**Tasiamide**	Several analogs	Linear peptides	*Symploca* sp.	KB; A549	IC_50_ = 0.8 to 8.5 µM IC_50_ = 2.24 to 12.88 μM		[92,93]
**Tiglicamides A–C**		Cyclic depsipeptides	*Lyngbya confervoides*	None: in vitro enzyme assay	IC_50_ = 2.14 to 7.28 µM	Serine protease inhibition (elastase)	[102]
**Ulongapeptin**		Cyclic depsipeptide	*Lyngbya* sp.	KB	IC_50_ = 0.63 µM.		[103]
**Veraguamides**	Veraguamides A–C and H–L veraguamides A–G	Cyclic depsipeptides	*Oscillatoria margaritifera* *Symploca* cf. *hydnoides*	NCI-H460; HT 29, HeLa	LD_50_ = 141 nM		[94,95]
**Wewakpeptins**		Depsipeptides	*Lyngbya semiplena*	NCI-H460	LD_50_ = 0.4 µM		[105]

## 3. Cyanobacteria as Nanoformulations in Cancer Therapies

Globally, the use of nanomaterials in the biomedical field has attracted the increasing interest of researchers because of their unique ability to interact with cells and tissues at the molecular level with a high degree of specificity and improved efficacy. The small size (ranging from 1 to 100 nm), design flexibility and large surface-to-volume ratio make these materials of significant use. With the appreciation of the enhanced properties of metals at nanosizes, extensive research on different nanometals has been carried out in recent years for exploring their applications in cancer research. Emphasizing the biological synthesis, cyanobacteria are considered as one of the best biological systems for nanoparticle (NP) synthesis, both intra-cellularly as well as extra-cellularly [111]. However, there are only a few reports available on the biological synthesis of noble metal NPs utilizing cyanobacteria as the host system. Silver (Ag) is the most commonly reported NP synthesized [111,112,113]. Besides this, gold (Au), palladium (Pd) and platinum (Pt) NPs of well-controlled size are also formed from cyanobacteria such as *Anabaena*, *Calothrix*, *Leptolyngbya*, etc. [113]. Moreover, several cyanobacterial strains have been used to synthesize selenium NPs (11.8 to 60 nm) [114]. Furthermore, *Spirulina subsalsa* has been utilized for the extracellular synthesis of Ag and Au NPs [115]. 

Very few studies on the above have suggested that these NPs could be used for the treatment of cancer (Table 2). Recently, silver NPs (AgNPs) synthesized from the aqueous extract of *Oscillatoria limnetica* have exhibited cytotoxic effects against human breast (MCF-7) and colon (HCT-116) cancer cell lines with IC_50_ values of 6.15 μg/mL and 5.37 μg/mL, respectively [116]. In another study, silver nanoparticles (20–50 nm size) synthesized from *L. majuscula* were screened against three leukemic cell lines, i.e., K562, MOLT-3, and REH, and showed dose- and time-dependent anticancer activity. The REH cells showed the maximum sensitivity to AgNPs, with an IC_50_ value of 620 ± 3.73 μg/mL [117]. Moreover, AgNPs (51–100 nm size) synthesized from the cell extract of the cyanobacterium *Nostoc* sp. strain HKAR-2 showed a dose-dependent cytotoxic activity against MCF-7 cancer cell lines, with an IC_50_ of 27.5 μg/mL [118]. The phycocyanin extracted from *Nostoc linckia* synthesizes silver nanoparticles (9.39 to 25.89 nm). These AgNPs exhibit in vitro antitumor activity against the human breast MCF-7 cancer cell line (IC_50_ = 27.79 ± 2.3 μg/mL), and were shown in vivo to inhibit tumor growth in Ehrlich ascites carcinoma-bearing mice [111]. ZnO NPs synthesized by using the cell extract of the *Nostoc* sp. EA03 exhibit less cytotoxicity when used in lower concentrations against the human adenocarcinoma alveolar basal epithelial cells (A549 cells) and human lung fibroblast (MRC-5 cells) cancer cell lines [119].

Cells and inhibited tumor growth. Consequently, they showed notable effects as anticancer agents and could be used as nanomedicines in cancer research. However, some cyanobacterial nanoformulations could also be used to deliver therapeutic drugs to cancer sites and monitor the tumor tissues, but limited data are available. As mentioned above, numerous studies show that, even at pico to nano molar concentrations, several cynobacterial secondary metabolites, such as apratoxin, calothrixin, curacin A, dolastatins, etc., are effective, and have shown potent inhibition of several cancer cell lines in vitro, however in vivo studies are limited. As with camptothecin, whose clinical application is limited due to its low solubility, although it has shown potent anticancer activity in vitro [120,121], most compounds isolated from cynobacteria have a low solubility in water, which makes their formulation troublesome or even incomprehensible [122,123]. Nanoformulation of cyanobacterial compounds represents an interesting strategy to overcome the hydrophobicity or low aqueous solubility of most natural bioactive compounds. Although further studies are needed to prove the concept, it can be hypothesized that if one of these compounds is combined with a nanomaterial, as stated above, such as gold, silver, zinc, etc., it could prove more effective. Furthermore, the use of nanomedicines has unique advantages over the systemic administration of free natural bioactive molecules. These advantages include the improved protection of the biological activities of the agents in a serum-rich environment, longer periods of circulation in the blood, improved permeability and effectiveness of tumor targeting, reduction of adverse effects and the possibility of considering reactivity to stimuli for personalized therapies, etc. [122,123,124]. Once encapsulated in nanoparticles, the hydrophobic bioactive compound becomes completely dispersible in water and can therefore be injected intravenously; the drug-loaded nanoparticles will then be able to release the active ingredient. However, only a very few studies have used this strategy with cyanobacterial compounds, which include nanovectorized extracts from *Arthrospira platensis* with anti-fungal biofilm activity [125] and the anti-inflammatory protein phycocyanin from *Aphanizomenon flosaquae* (a freshwater cyanobacteria usually known as AFA Klamath), the delivery of which to the deep skin layers was improved when using propylene glycol-containing nanovesicles [126]. However, no attempt to nanovectorise a cyanobacterial compound with an anticancer effect has been reported to date and this would merit future research.

**Table 2:** Anticancer potential of some nanoparticles synthesized from different cyanobacterial strains.

Cyanobacterial Strain	Type of Nanoparticles	Cancer Cell Lines	IC_50_ Value	References
*Oscillatoria limnetica*	AgNPs	MCF-7, HCT-116	6.15 and 5.37 μg/mL	[111]
*Lyngbya majuscula*	AgNPs	K562, MOLT-3, REH	620 ± 3.73 μg/mL	[119]
*Nostoc* sp. strain HKAR-2	AgNPs	MCF-7	27.5 μg/mL	[124]
*Nostoc linckia*	AgNPs	MCF-7	27.79 ± 2.3 μg/mL	[127]
*Nostoc* sp. EA03	ZnO	A549, MRC-5		[127]

Previously, green carbon nanotags (G-tags) developed from cyanobacteria were shown to be an advanced and efficient imaging platform for anticancer therapy due to their high solubility, excellent photostability and low cytotoxicity. These nanotags, when conjugated with doxorubicin, not only induced cell death in cancer cells (HepG2 and MCF-7), but also, the fluorescence of G-tags enables the monitoring of doxorubicin uptake by cancer cells and their intracellular location [127]. Similar studies could also be carried forward by conjugating cynobacterial metabolites with nanotags, or the nanovectorization of canonical anticancer drugs using cynobacteria to enhance the anticancer potential of the drug. Cynobacterial metabolites from *Spirulina* showed protective effects against the cardiotoxicity induced by doxorubicin, and thus improve the therapeutic index of doxorubicin [128]. Similarly, the biochemical protective effects of *Spirulina platensis* against oxidative stress caused by doxorubicin were also evaluated [129]. These could find future applicability, with a better shelf-life and stability as natural capping occurs [130].

## 4. Conclusions

In the last few decades, the use of cyanobacteria as a novel source of therapeutics has been realized. Several chemically diverse compounds have been screened against different cancer cell lines, but still, very few cyanobacterial compounds have entered clinical trials, as much is not known at the molecular level. Though their economic cultivation expanded their utilization in drug discovery, still there is a need to explore more deeply into cyanobacterial compounds in order to clarify the specific targets and the mechanisms involved in cancer. Thus, more scientific attention and interdisciplinary research would have to be devoted to finding novel compounds by exploring cyanobacterial strains from extreme unexplored habitats. Furthermore, we do not explore in this review paper the structure–function relationship that might be established and would link a structural motif to a particular target. To this end, the reader may refer to the comprehensive review published previously by Salvador-Reyes and Luesch [131], which details the mechanisms of action of cyanobacterial metabolites alongside the methodology used for these discoveries. Besides this, Xu et al. focused on calothrixins synthesis and their biological effects [27]. For example, quinone compounds such as calothrixin A and B are known to be redox-active, and the generation of ROS through redox cycling of the quinone was thought to be responsible of the induced apoptosis and DNA damage [27]. Structure–function studies showed that this mechanism of action was independent of the presence of calothrixin’s ring E, but strongly dependent on the tetracyclic ring quinone structure [27] Further analyses highlighted the crucial role of the nitrogen in ring D, the importance of ring A–D, and finally evidenced the higher potential of calothrixin B [27].

One of the main subjects of interest in the field of natural biomedicines and their nanoformulations is their toxicity and/or that of contaminating toxic substances. Indeed, cyanobacteria could also be the sources of toxin production [86]. Although generally free of contaminants, cyanobacterial products may contain substances harmful to the liver, such as microcystins, toxic metals and other types of harmful bacteria, which are dangerous for human consumption [132]. Contaminated cyanobacteria can cause liver damage, stomach pain, nausea, vomiting, weakness, thirst, rapid heartbeat, shock and even death. The presence of microcystins is well known for its hepatotoxicity [132], and several other compounds cause severe anaphylaxis and neuromuscular toxicity [133]. This biosafety issue may partly explain the very low number of cyanobacterial compounds reaching clinical trials, mainly Brentuximab vedotin for Hodgkin lymphoma and Glembatumumab vedotin for various cancers (Table S1) [6]. Although this is also true for numerous other compounds that exhibit strong anticancer activity but did not reach clinical trial, this might be due to their toxic nature, which hindered the biological activities in vivo or their natural contamination with toxicants while processing the drug.

In addition, the biosynthesis of NP by cyanobacteria is a relatively new idea to which promising results are attributed and, in the future, it will be possible to build datasets for marine pharmaceuticals by developing nanoformulated drugs based on cyanobacteria. However, to bring it to the platform of drug development there is a need to optimize biosynthesis pathways involving the futuristic approach of green synthesis by controlling the size and shape of particles and achieving monodispersity in the solution phase. As cyanobacteria act as natural therapeutic and have pharmaceutical potential, it could be expected that they could be used as an antitumor killer for a better world.

## Supplementary Materials

The following are available online. Table S1: Clinical trials involving cyanobacterial-derived compounds.
