# Supplementary material for: Cyanobacteria as Natural Therapeutics and Pharmaceutical Potential: Role in Antitumor Activity and as Nanovectors
Source: Molecules. 2021 Jan 5;26(1):247. doi: 10.3390/molecules26010247 (PMC7796498; doi:10.3390/molecules26010247)
Supplement: Supplementary file 1 [file molecules-26-00247-s001.pdf]

Table S1: Clinical trials involving cyanobacterial-derived compounds, which are recruiting, completed or terminated.

NCT Number[a]	Official Title	Conditions	Phase	Study Design	Start Date and End Date	Responsible, Country
NCT03664323	Efficacy of Chemotherapy or Chemo-anti-PD-1 Combination After Failed Anti-PD-1 Therapy for Relapsed and Refractory Hodgkin Lymphoma: a Series From Lysa Centers.	Hodgkin Lymphoma	n.a.[b]	Observational Model: Cohort (30 individuals)<br>Time Perspective: Retrospective	January 2018<br>Completed June 2018	Hospices Civils de Lyon, France
NCT02098512	A Multicenter Pilot Study of Reduced Intensity Conditioning and Allogeneic Stem Cell Transplantation Followed by Targeted Immunotherapy in Children, Adolescents and Young Adults with Poor Risk CD30+ Hodgkin Lymphoma (HL)	Hodgkin Lymphoma	Phase I<br>Phase II	Intervention Model: Single Group Assignment<br>Masking: None (Open Label)<br>Primary Purpose: Treatment	March 2014<br>recruiting	New York Medical College, USA
NCT02713828	A Phase I/II Study of Glembatumumab Vedotin in Patients With gpNMB-Expressing, Advanced or Metastatic Squamous Cell Carcinoma of the Lung	Squamous Cell Carcinoma of the Lung	Phase I<br>Phase II	Allocation: Non-Randomized<br>Intervention Model: Parallel Assignment<br>Masking: None (Open Label)<br>Primary Purpose: Treatment	April 2016<br>Terminated September 2018	PrECOG, LLC, USA<br>Several Institutions in the USA
NCT02487979	A Phase 2 Study of GPNMB-Targeted Antibody-Drug Conjugate, CDX-011 (Glembatumumab Vedotin, CR011-vcMMAE; NSC# 763737), in Recurrent or Refractory Osteosarcoma	Recurrent Osteosarcoma	Phase II	Allocation: N/A<br>Intervention Model: Single Group Assignment<br>Masking: None (Open Label)<br>Primary Purpose: Treatment	February 2016<br>Completed June 2017	National Cancer Institute, USA
NCT02302339	A Phase 2 Study of Glembatumumab Vedotin, an Anti-gpNMB Antibody-drug Conjugate, as Monotherapy or in Combination With Immunotherapies in Patients With Advanced Melanoma	Melanoma	Phase II	Allocation: Non-Randomized<br>Intervention Model: Single Group Assignment<br>Masking: None (Open Label)<br>Primary Purpose: Treatment	November 2014<br>Terminated October 2018	Celldex Therapeutics, USA
NCT02363283	A Phase 2 Study of CDX-011 (Glembatumumab Vedotin) for Metastatic Uveal Melanoma	Recurrent Uveal Melanoma;<br>Stage IV Uveal Melanoma AJCC v7	Phase II	Allocation: N/A<br>Intervention Model: Single Group Assignment<br>Masking: None (Open Label)<br>Primary Purpose: Treatment	September 2015<br>Completed July 2018	National Cancer Institute, USA
NCT01997333	A Randomized Multicenter Pivotal Study of CDX-011 (CR011-vcMMAE)in Patients With Metastatic, gpNMB Over-Expressing, Triple Negative Breast Cancer (The METRIC Study)	Metastatic gpNMB Over-expressing Triple Negative Breast Cancer	Phase II	Allocation: Randomized<br>Intervention Model: Parallel Assignment<br>Masking: None (Open Label)<br>Primary Purpose: Treatment	November 2013<br>Completed August 2018	Celldex Therapeutics, USA

NCT01156753	A Phase II, Randomized, Multicenter Study of CDX-011 (CR011-vcMMAE) in Patients With Advanced GPNMB-expressing Breast Cancer	Breast Cancer	Phase II	Allocation: Randomized<br>Intervention Model: Parallel Assignment<br>Masking: None (Open Label)<br>Primary Purpose: Treatment	July 2010<br><br>Completed<br>November 2012	Celldex Therapeutics, USA
NCT00412828	A Phase I/II Study of CR011-vcMMAE in Subjects With Unresectable Stage III or Stage IV Melanoma	Unresectable Stage III or Stage IV Melanoma	Phase I<br>Phase II	Allocation: N/A<br>Intervention Model: Single Group Assignment<br>Masking: None (Open Label)<br>Primary Purpose: Treatment	June 2006<br><br>Completed<br>May 2011	Celldex Therapeutics, USA
NCT00704158	A Phase I/II Study of CR011-vcMMAE in Patients With Locally Advanced or Metastatic Breast Cancer	Breast Cancer	Phase I<br>Phase II	Allocation: N/A<br>Intervention Model: Single Group Assignment<br>Masking: None (Open Label)<br>Primary Purpose: Treatment	June 2008<br><br>Completed<br>June 2011	Celldex Therapeutics, USA

[a] NCT, Number Clinical Trials (identifier); b n.a., not applicable. Source "CinicalTrials.gov" database [1].

1. ClinicalTrials.gov is a database of privately and publicly funded clinical studies conducted around the world. It is a resource provided by the United States National Library of Medicine. Available online: https://clinicaltrials.gov/ (accessed on December 10th 2020).
